# Neurodevelopmental and synaptic defects in *DNAJC6* parkinsonism, amenable to gene therapy

**DOI:** 10.1093/brain/awae020

**Published:** 2024-01-18

**Authors:** Lucia Abela, Lorita Gianfrancesco, Erica Tagliatti, Giada Rossignoli, Katy Barwick, Clara Zourray, Kimberley M Reid, Dimitri Budinger, Joanne Ng, John Counsell, Arlo Simpson, Toni S Pearson, Simon Edvardson, Orly Elpeleg, Frances M Brodsky, Gabriele Lignani, Serena Barral, Manju A Kurian

**Affiliations:** Developmental Neurosciences, Zayed Centre for Research into Rare Disease in Children, UCL Great Ormond Street Institute of Child Health, London, WC1N 1DZ, UK; Developmental Neurosciences, Zayed Centre for Research into Rare Disease in Children, UCL Great Ormond Street Institute of Child Health, London, WC1N 1DZ, UK; Department of Clinical and Experimental Epilepsy, UCL Queen Square Institute of Neurology, University College London, London, WC1N 3BG, UK; Laboratory of Pharmacology and Brain Pathology, Humanitas Clinical and Research Center, 20089 Milano, Italy; Developmental Neurosciences, Zayed Centre for Research into Rare Disease in Children, UCL Great Ormond Street Institute of Child Health, London, WC1N 1DZ, UK; Developmental Neurosciences, Zayed Centre for Research into Rare Disease in Children, UCL Great Ormond Street Institute of Child Health, London, WC1N 1DZ, UK; Developmental Neurosciences, Zayed Centre for Research into Rare Disease in Children, UCL Great Ormond Street Institute of Child Health, London, WC1N 1DZ, UK; Department of Clinical and Experimental Epilepsy, UCL Queen Square Institute of Neurology, University College London, London, WC1N 3BG, UK; Developmental Neurosciences, Zayed Centre for Research into Rare Disease in Children, UCL Great Ormond Street Institute of Child Health, London, WC1N 1DZ, UK; Developmental Neurosciences, Zayed Centre for Research into Rare Disease in Children, UCL Great Ormond Street Institute of Child Health, London, WC1N 1DZ, UK; Developmental Neurosciences, Zayed Centre for Research into Rare Disease in Children, UCL Great Ormond Street Institute of Child Health, London, WC1N 1DZ, UK; Genetic Therapy Accelerator Centre, UCL Queen Square Institute of Neurology, London, WC1N 3BG, UK; Developmental Neurosciences, Zayed Centre for Research into Rare Disease in Children, UCL Great Ormond Street Institute of Child Health, London, WC1N 1DZ, UK; Developmental Neurosciences, Zayed Centre for Research into Rare Disease in Children, UCL Great Ormond Street Institute of Child Health, London, WC1N 1DZ, UK; Department of Neurology, Columbia University Irving Medical Center, New York, NY 10032-3784, USA; Department of Pediatrics, Nationwide Children’s Hospital, Ohio State University, Columbus, OH 43210, USA; Department of Neurology, Nationwide Children’s Hospital, Ohio State University, Columbus, OH 43210, USA; Department of Genetics, Hadassah, Hebrew University Medical Center, 9574869 Jerusalem, Israel; Department of Genetics, Hadassah, Hebrew University Medical Center, 9574869 Jerusalem, Israel; Research Department of Structural and Molecular Biology, Division of Biosciences, University College London, London, WC1E 6BT, UK; Developmental Neurosciences, Zayed Centre for Research into Rare Disease in Children, UCL Great Ormond Street Institute of Child Health, London, WC1N 1DZ, UK; Department of Clinical and Experimental Epilepsy, UCL Queen Square Institute of Neurology, University College London, London, WC1N 3BG, UK; Developmental Neurosciences, Zayed Centre for Research into Rare Disease in Children, UCL Great Ormond Street Institute of Child Health, London, WC1N 1DZ, UK; Developmental Neurosciences, Zayed Centre for Research into Rare Disease in Children, UCL Great Ormond Street Institute of Child Health, London, WC1N 1DZ, UK; Department of Neurology, Great Ormond Street Hospital, London, WC1N 3JH, UK

**Keywords:** DNAJC6, auxilin, parkinsonism, CME, neurodevelopmental, gene therapy

## Abstract

*DNAJC6* encodes auxilin, a co-chaperone protein involved in clathrin-mediated endocytosis (CME) at the presynaptic terminal. Biallelic mutations in *DNAJC6* cause a complex, early-onset neurodegenerative disorder characterized by rapidly progressive parkinsonism-dystonia in childhood. The disease is commonly associated with additional neurodevelopmental, neurological and neuropsychiatric features. Currently, there are no disease-modifying treatments for this condition, resulting in significant morbidity and risk of premature mortality.

To investigate the underlying disease mechanisms in childhood-onset *DNAJC6* parkinsonism, we generated induced pluripotent stem cells (iPSC) from three patients harbouring pathogenic loss-of-function *DNAJC6* mutations and subsequently developed a midbrain dopaminergic neuronal model of disease.

When compared to age-matched and CRISPR-corrected isogenic controls, the neuronal cell model revealed disease-specific auxilin deficiency as well as disturbance of synaptic vesicle recycling and homeostasis. We also observed neurodevelopmental dysregulation affecting ventral midbrain patterning and neuronal maturation. To explore the feasibility of a viral vector-mediated gene therapy approach, iPSC-derived neuronal cultures were treated with lentiviral *DNAJC6* gene transfer, which restored auxilin expression and rescued CME.

Our patient-derived neuronal model provides deeper insights into the molecular mechanisms of auxilin deficiency as well as a robust platform for the development of targeted precision therapy approaches.

## Teaser

Abela *et al.* identify neurodevelopmental and synaptic vesicle recycling defects in a patient-derived neuronal cell model of early-onset *DNAJC6* parkinsonism. Rescue of key cellular disease features using lentiviral *DNAJC6* delivery demonstrates the utility and feasibility of a potential gene therapy approach.

## Introduction

Disturbance of synaptic sorting and trafficking is increasingly linked to a broad spectrum of neurological disorders. Clathrin-mediated endocytosis (CME) is a key mechanism in synaptic vesicle recycling and is involved in crucial synaptic and developmental signalling processes.^1^ Dysregulation of CME has been linked to a variety of human diseases, in particular neurological disorders^1^; mutations in genes encoding key CME proteins (*DNAJC6*, *CLTC*, *SYT* and *SYNJ1*) have been described in patients with complex neurological syndromes presenting with intellectual disability, movement disorders and epilepsy.^2-11^ Patients with biallelic *DNAJC6* mutations develop parkinsonian symptoms in late childhood or early adolescence, with rapid neurological decline and loss of ambulation.^2-6^ The majority of patients with this juvenile form of disease present with early neurodevelopmental delay and intellectual disability that usually precedes the movement disorder.^3,5,6^ The disease course is often further complicated by seizures and neuropsychiatric symptoms. In contrast, patients with later-onset *DNAJC6-*disease present with parkinsonism in the third or fourth decade of life.^4^ Genotype-phenotype correlations are now emerging: patients with rapidly progressive juvenile-onset disease typically harbour recessive loss-of-function or early splice site mutations,^2-6^ whereas patients with later-onset disease commonly have missense changes or late splicing variants associated with a milder disease course and better response to treatment.^4^

**Figure 1:**
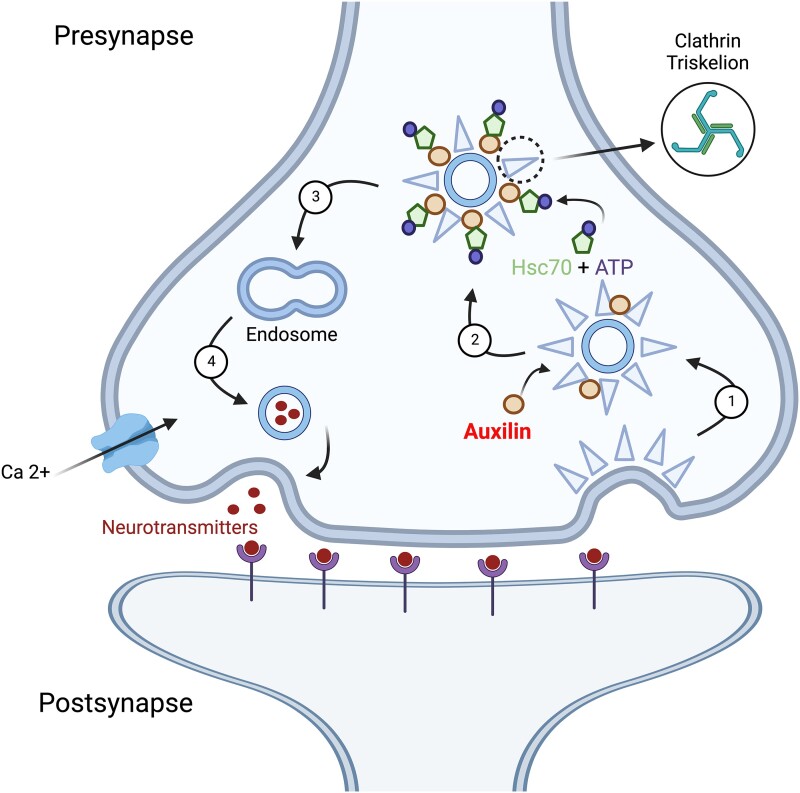
**Schematic representation of CME.** Clathrin-mediated endocytosis with (**1**) nucleation of clathrin-coated vesicles and clathrin coat assembly; (**2**) uncoating and release of cargo; (**3**) endolysosomal recycling of synaptic vesicles (SV); and (**4**) synaptic vesicle exocytosis. CME = clathrin-mediated endocytosis. Created with BioRender.com.

*DNAJC6* encodes auxilin, a co-chaperone protein of the DNAJ family. Auxilin is a neuronal-specific protein enriched in presynaptic terminals, where it contributes to CME (Fig. 1). CME requires a machinery of distinct presynaptic proteins that interact in several steps,^12-16^ namely (i) nucleation of clathrin-coated pits by clathrin assembly at adaptor complexes; (ii) cargo uptake into clathrin-coated vesicles through recruitment by adaptors; (iii) clathrin coat disassembly (uncoating); and (iv) delivery of vesicle contents to the endosome from where synaptic vesicles are generated. In an overall process called synaptic vesicle recycling, synaptic CME recaptures synaptic vesicle proteins that have been released during neurotransmission for repackaging. Clathrin coats have also been implicated at the endosomal synaptic vesicle formation step.^17^ Auxilin is involved in clathrin uncoating and binds to clathrin in a ratio of 1:3, then subsequently recruits the chaperone protein Hsc70 via its J-domain to trigger Hsc70-ATPase activity. Hydrolysis of ATP finally results in clathrin coat distortion and disassembly. Synaptic vesicles are recycled via the endosomal compartment. A growing body of evidence shows that dysfunction of synaptic vesicle recycling is involved in the pathogenesis of Parkinson’s disease (PD).^18^ Studies in both monogenic and sporadic parkinsonism have revealed that proteins encoded by specific PD-causing (*SYNJ1*, *SNCA*, *PRKN*) and PD-associated genes (*LRRK2*, *SH3GL2*) are involved in key steps of synaptic vesicle recycling and its regulation.^19-21^ Disruption of synaptic vesicle membrane traffic affects synaptic transmission, autophagy and the endolysosomal system and causes axon degeneration and ultimately dopaminergic neurodegeneration.^18,22^

Patients with recessive juvenile-onset *DNAJC6-*associated disease have loss-of-function mutations, which result in auxilin deficiency. Previous studies have shown that in patients with biallelic *DNAJC6* mutations, auxilin levels were significantly reduced, both in fibroblasts and CSF.^6^^123^I-N-ω-fluoropropyl-2β-carbomethoxy-3β-(4-iodophenyl) nortropane single photon emission computed tomography (^123^I-FP-CIT SPECT) showed reduced tracer uptake in the basal ganglia (suggestive of presynaptic striatonigral neurodegeneration) and significantly reduced presynaptic dopaminergic proteins in patient CSF, indicating disruption of dopamine homeostasis.^6^ Auxilin deficiency has also been investigated in several animal models. An auxilin knockout mouse model showed accumulation of clathrin-coated vesicles (CCVs) and empty clathrin cages at the presynaptic terminal with subsequent impairment of synaptic vesicle recycling.^23^ A *Drosophila* auxilin knockdown model exhibited reduced locomotion and longevity, with age-dependent loss of dopaminergic neurons.^24^

In this study, we sought to better define the pathogenic mechanisms that underpin *DNAJC6*-related parkinsonism by developing a humanized midbrain dopaminergic neuron (mDA) cellular model generated from patient-derived induced pluripotent stem cells (iPSC).

## Materials and methods

### Generation and maintenance of human iPSC lines

Stored fibroblasts from patients with *DNAJC6-*related disease were used for this study; written informed consent was obtained from all participating families and the study was approved by local ethics committees (Reference 13/LO/0171, Columbia University IRB-AAAA7128, Reference 0393-17-HMO). Age-matched control fibroblasts were obtained from the MRC Centre for Neuromuscular Disorders Biobank. All human fibroblasts were tested for mycoplasma infection prior to use. Human dermal fibroblasts were maintained in supplemented Dulbecco’s modified Eagle medium (DMEM, Gibco). Human iPSCs (hiPSCs) were generated according to an adapted protocol from the CytoTune™ iPS 2.0 Sendai Reprogramming Kit (Invitrogen), as previously described.^25^ All iPSC lines were thoroughly characterized for genomic integrity and pluripotency (Supplementary material). Two age-matched control lines from a healthy donor (Control 1, Control 2) have already been established and characterized previously in our laboratory.^25,26^ An isogenic control line for Patient 1 was generated by CRISPR-Cas9 correction of the c.766C>T variant (Supplementary material).

### Differentiation of midbrain dopaminergic neurons

Control and patient mDA-derived lines were differentiated from iPSCs, as previously described.^25^ In summary, cells were harvested with TrypLE™ (Invitrogen) and plated on non-adherent dishes in DMEM/F12:Neurobasal (1:1), N2 (1:100) and B27 minus vitamin A (1:50) supplements (Invitrogen), 2 mM L-glutamine, 1% penicillin/streptomycin (P/S) and ROCK-inhibitor from Day 0 to Day 2 to allow embryoid body formation. Embryoid bodies were collected at Day 4 and plated on 12-well plates previously coated with polyornithine (PO; 15 μg/ml; Sigma), fibronectin (FN; 5 μg/ml Gibco) and laminin (LN; 5 μg/ml; Sigma). Day 0 to Day 9 medium was supplemented with: 10 μM SB431542 (Tocris Bioscience), 100 nM LDN193189 (Stemgent Inc.), 0.8 µM CHIR99021 (Tocris Biosceince) and 100 ng/ml hSHH-C24-II (R&D Systems). On Day 2, 0.5 μM purmorphamine (Cambridge Bioscience) was added and SB431542 was withdrawn at Day 6. On Day 11, the cells were either analysed to check gene expression of mDA precursors markers or harvested with Accumax™ and plated in droplets (1–1.5 × 10^4^ cells/µl) on pre-coated (PO/FN/LN) plates. From Day 11 onwards, the medium was composed as follows: neurobasal B27 minus vitamin A (1:50), 2 mM L-glutamine, P/S, 0.2 mM ascorbic acid and 20 ng/ml brain-derived neurotrophic factor (BDNF, Miltenyi Biotech) and supplemented on Day 14 of differentiation with 0.5 mM dibutyryl c-AMP (Sigma-Aldrich) and 20 ng/ml glial cell line-derived neurotrophic factor (GDNF, Miltenyi Biotech). On Day 30 of differentiation, cells were replated, as described earlier, onto PO/FN/LN-coated dishes or Labtek slides (Nunc™) and γ-secretase inhibitor DAPT (10 μM, Tocris) was added until collection at Day 65–70.

### FM™1–43 dye uptake assay

Differentiated 70-day-old mDA neurons, seeded on Labtek slides (Nunc™), were incubated with Hanks’ balanced salt solution (HBSS) medium (Thermo Fisher) supplemented with 2 mM Ca^2+^ and 2 mM Mg^2+^ for 10 min at 37°C. The medium was changed to HBSS medium (Thermo Fisher) supplemented with 2 mM Ca^2+^, 2 mM Mg^2+^, FM™1–43 × (Thermo Fisher) 5 µg/ml, NBQX (Tocris Biosciences) 10 µM and KCl 60 mM for 2 min at 37°C. The cells were then incubated with HBSS medium (Thermo Fisher) supplemented with 2 mM Ca^2+^, 2 mM Mg^2+^ and FM™1–43 × 5 µg/ml for 15 min at 37°C. mDA neurons were then carefully washed with HBSS without Ca^2+^ and Mg^2+^ (Thermo Fisher) and fixed at room temperature for 10 min with 4% paraformaldehyde (PFA) diluted in HBSS medium without Ca^2+^ and Mg^2+^. Neuronal cultures were then imaged using the confocal microscope Zeiss LSM710 and processed using ImageJ software (National Institutes of Health). Mean fluorescence intensity of synaptic boutons was quantified as an average from 15 randomly selected regions of interest (ROIs) in four images from three independent differentiations from each donor line.

### Electron microscopy analysis

Differentiated 65–70-day-old iPSCs were fixed with 2% PFA and 1.5% glutaraldehyde in 0.1 M phosphate buffer pH 7.3, postfixed in 1% OsO_4_, 1.5% K_4_Fe(CN)_6_, and 0.1 M sodium cacodylate, dehydrated and flat embedded in Araldite resin (Araldite CY212, Agar Scientific). Ultrathin sections (70 nm) were collected on copper mesh grids (EMS) and observed with a Jeol 1400 Flash transmission electron microscope at 100 kV equipped with a Gatan RIO camera (Gatan). Morphometric analysis was done using ImageJ (NIH). Structures with sagittal diameter comprised between 20 and 60 nm were classified as synaptic vesicles, while those with a sagittal diameter bigger than 60 nm were classified as intra-terminal cisternae. Synaptic vesicles touching the active zone were classified as docked synaptic vesicles.

### Bulk RNA sequencing analysis

Total RNA was isolated using the RNeasy® mini kit (Qiagen) following manufacturer’s instructions. RNA libraries were prepared from 100 ng of total RNA using KAPA mRNA HyperPrep kit (Roche) according to the manufacturer’s protocol and sequenced with Illumina NextSeq 500/550 High Output single-end (∼30 M reads/sample). FASTQ obtained files were uploaded to and analysed on Galaxy web platform (usegalaxy.org).^27^ FASTQ files were filtered with fastp (v.0.20.1),^28^ trimming per quality (mean quality > Q20) and discarding low quality (phread quality >15) reads. Mapping to human reference genome (GRCh38) was performed with HISAT2 (v.2.1.0)^29^ and counts for genes were extracted with featureCounts (v.1.6.4) excluding duplicates, multimapping reads and chimeric fragments.^30^ Differential gene expression was analysed using edgeR (v.3.24.1),^31^ filtering low counts at one minimum counts per million, in at least three samples^32^ and comparing disease status (patients versus control) and disease-corrected genotype (Patient 1 versus CRISPR Patient 1). Differentially expressed genes (DEGs) with a false discovery rate correction (FDR) < 0.05 and absolute fold change >2 were considered as statistically significant and protein-codifying DEGs were considered for further analyses. Heat maps were generated from the row-scaled *z*-score of normalized counts obtained by EdgeR with complete-linkage Euclidean hierarchical clustering. Gene ontology (GO) enrichment analyses were performed using PANTHER v14^33^ for biological process, Enrichr for cellular process and SynGO (v.1.1)^34^ for synaptic localization, with Fisher’s exact test correction of FDR < 0.05. Raw data and results were deposited in GEO (Gene Expression Omnibus), under accession number GSE208353.

### Statistical analysis

GraphPad Prism version 8 was used for statistical analysis. Unpaired two-tailed Student’s *t*-test was applied for single comparisons and one-way ANOVA followed by Tukey’s multiple comparisons test were performed for multiple comparisons. A paired two-tailed Student’s *t*-test was use for the FM-43 uptake analysis. Data are presented as mean ± standard error mean (SEM) from at least three independent differentiations from each donor line with no sample pooling. The exact number for each experiment is clarified in the corresponding figure legend. Significance levels are defined by *P*-values and indicated with asterisks on graphs. *P*-values are shown as *P = 0.05–0.01, **P = 0.01–0.001 and ***P < 0.001.

## Results

### Generation of a patient-derived midbrain dopaminergic neuronal cell model

We developed iPSC lines from dermal fibroblasts taken from three patients with loss-of-function homozygous *DNAJC6* mutations: Patient 1: c.766C>T, p.R256*, Patient 2: c.2416C>T, p.R806* and Patient 3: c.801-2 A>G. The phenotype of the patients is summarized in Table 1. Patient and control iPSC lines have been developed and characterized, as previously described.^25,26^ Retention of the patient-specific *DNAJC6* mutations was confirmed by Sanger sequencing (Supplementary Fig. 1A). All iPSC lines were evaluated for genomic integrity, clearance of Sendai virus transgenes and true pluripotency characteristics (Supplementary Figs 1B and C and 2A–D). IPSCs were subsequently differentiated into mDA neurons and fully characterized at Day 11 and Day 65 for ventral midbrain and mature neuronal markers (Supplementary Fig. 3A and B).

**Table 1:** Information on genotype and phenotype of three patients with biallelic *DNAJC6* mutations

	Patient 1	Patient 2	Patient 3
**Genotype**			
Inheritance	Recessive	Recessive	Recessive
*DNAJC6* mutation/protein effect	c.766C>T/p.R256*	c.2416C>T/p.R806*	c.801-2A>G/-
Predicted effect	Protein truncating	Protein truncating	Splice site
**Movement disorder phenotype**			
Onset of parkisonism (years)	13	10	11
Bradykinesia	+	+	+
Tremor	+	+	+
Rigidity	+	+	+
Hypomimia	+	+	+
Postural instability	+	+	+
Dysarthria	Anarthric	+	+
Loss of ambulation (years)	13	15	13
Motor fluctuations	−	+	
Response to levodopa/carbidopa	Unsustained (5.5 mg/kg/day)	Some response (200 mg/day)	No response
Other effective medications	−	Trihexyphenidyl	−
**Neurodevelopmental phenotype**			
Early development	Delayed	Delayed	Normal
Cognition	Cognitive impairment	Cognitive impairment	Normal
**Additional neurological features**			
Seizures	Generalized	Generalized	−
Dystonia	+	+	−
Myoclonus	+	−	−
Psychiatric features	Anxiety, sleep disorder	Attention deficit, aggressive behaviour	−

+ = present; − = absent.

### Disease-specific defects in synaptic vesicle recycling, homeostasis and neurotransmission

**Figure 2:**
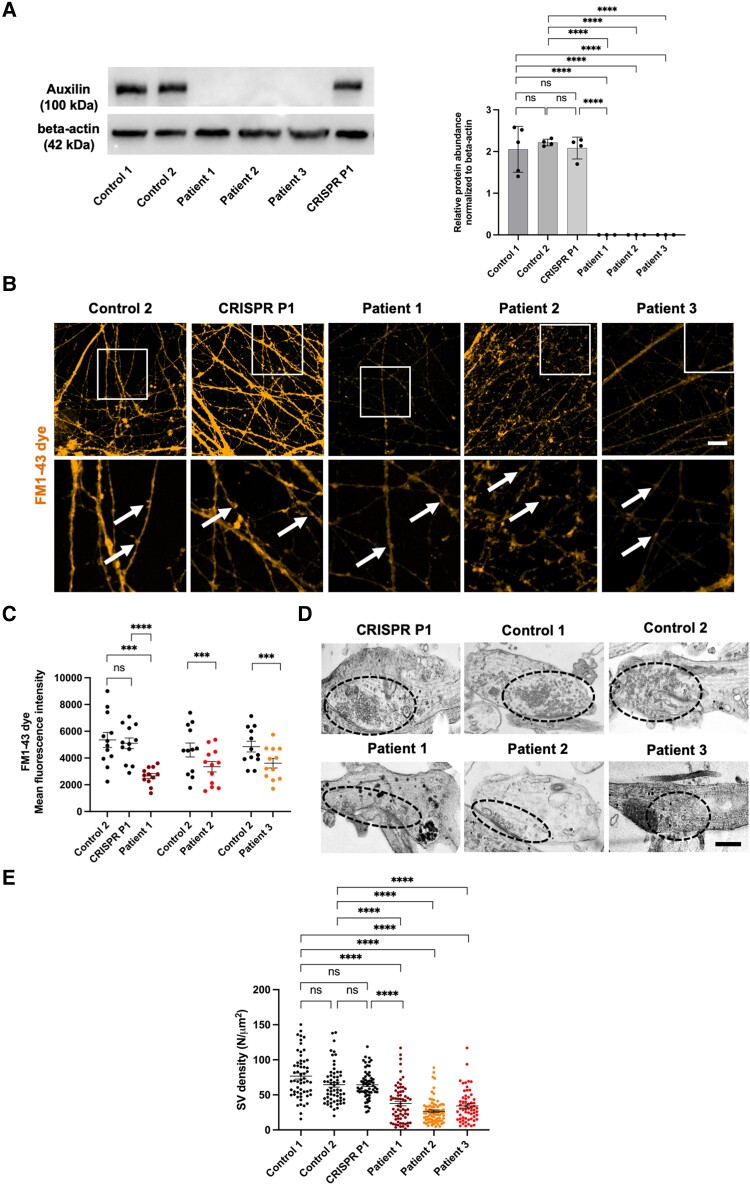
**Patient-derived neurons show auxilin deficiency and disturbance of CME and synaptic vesicle homeostasis.** (**A**) Immunoblot analysis for auxilin protein in control, patient and CRISPR Patient P1-derived neurons at Day 65 of differentiation. Quantification of protein abundance relative to the loading control (β-actin) (auxilin *n* = 5, 4, 4, 3, 3, 3). (**B**) Representative images of FM1–43 dye uptake assay for control, patient and CRISPR Patient P1-derived neurons at Day 65 of differentiation. Arrows in the high magnification images (*bottom row*) show representative synaptic boutons. Scale bar = 20 μm. (**C**) Quantification of FM1–43 mean fluorescence intensity in patients relative to the control and Patient 1 versus CRISPR Patient P1, respectively. (**D**) Electron microscopy analysis of presynaptic terminals in control, patient and CRISPR Patient P1-derived neuronal cultures at Day 65 of differentiation. Scale bar = 500 nm. Dotted circles indicate area with synaptic vesicles. (**E**) Quantification of synaptic vesicle (SV) density in the presynaptic terminal on electron microscopy images. CME = clathrin-mediated endocytosis; ns = not significant.

To confirm that our patient-derived neuronal model recapitulates key molecular disease phenotypes, we first evaluated the effect of biallelic *DNAJC6* mutations on auxilin protein expression in mDA neuronal populations. Whilst auxilin was present in the age-matched control, it was absent in all patient lines, but notably fully restored in the isogenic control line (Fig. 2A). Auxilin binds with high affinity to the C-terminal of the clathrin heavy chain (CHC) subunit to initiate the uncoating process.^13^ To determine the effect of biallelic *DNAJC6* mutations on CME, we performed an FM1–43 uptake assay to monitor membrane uptake during synaptic vesicle recycling.^35^ FM1–43 dye is characterized by a water-soluble polar head and a lipid-soluble hydrophobic tail, separated by a central region that defines the spectral properties of the dye.^36^ In aqueous solutions, FM1–43 is non-fluorescent, but when bound to membrane lipids by its hydrophobic tail, it becomes brightly fluorescent. FM1–43 dye inserts into the outer leaflet of the cell surface membrane and is internalized within the inner membrane of nascent synaptic vesicles, thereby visualizing the process of endocytosis. Intracellular uptake of the FM™1–43 dye was significantly reduced in all patient mDA lines indicating impairment of CME at the presynaptic terminals (Fig. 2B and C). We then performed electron microscopy analysis to assess the effect of impaired CME on synaptic vesicle homeostasis. We observed a significantly reduced number of synaptic vesicles in the presynaptic terminal in all patient mDA lines (Fig. 2D and E), while other synaptic parameters were not consistent across patient and control lines. Synaptic area remained unchanged in Patient 1, while active zone length was significantly increased (versus Control 2)/unchanged (versus Control 1) and the number of docked synaptic vesicles per active zone length significantly reduced, both of which were restored in the isogenic control (Supplementary Fig. 4). In Patient 2, both synaptic area and active zone length were increased (versus Control 2)/unchanged (versus Control 1), while docked synaptic vesicles per active zone length was reduced. In Patient 3, no significant differences were observed except for reduced active zone length (versus Control 1) (Supplementary Fig. 4). We then wanted to evaluate the effect of reduced synaptic vesicle numbers on synaptic neurotransmission. We recorded spontaneous excitatory post-synaptic currents (sEPSCs) in Patient 1 and its isogenic control and found similar resting membrane potential and frequency of events, but a significantly reduced amplitude of events in Patient 1 compared to CRISPR Patient P1 (Supplementary Fig. 5A and B).

### Disease-specific aberrant early ventral midbrain patterning and impaired neuronal maturation

**Figure 3:**
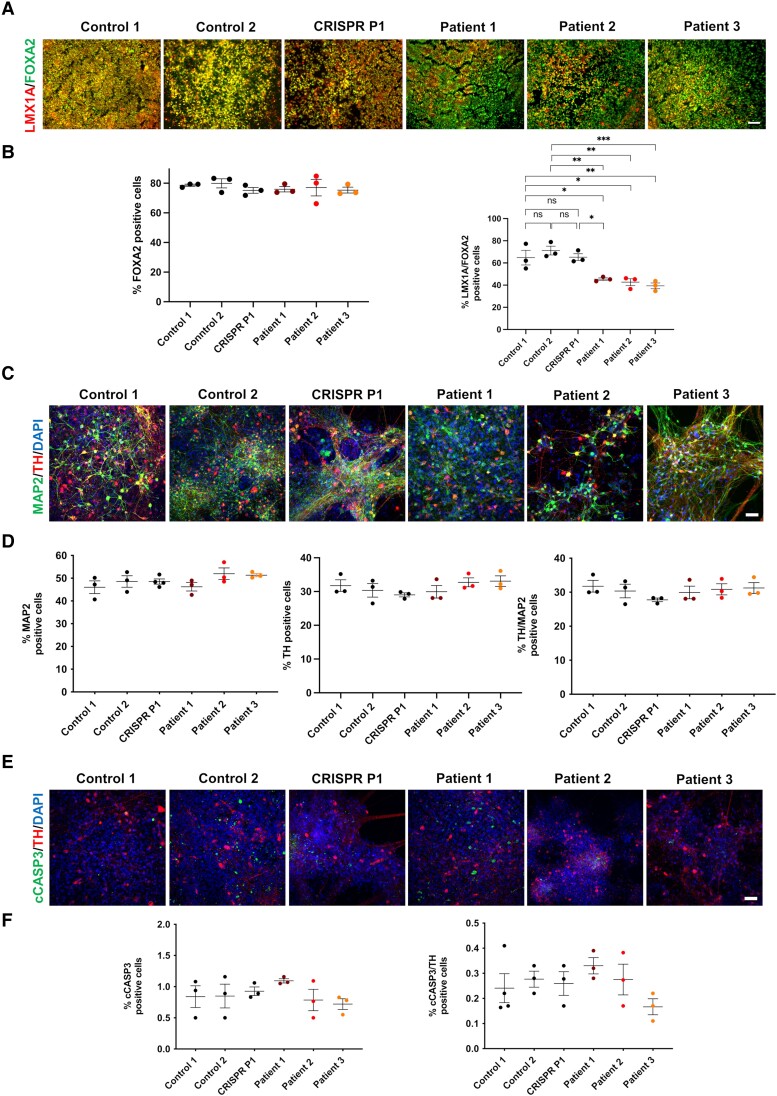
**Patient-derived neurons show alterations in ventral midbrain patterning without neurodegeneration.** (**A**) Representative immunofluorescence images for LMX1A and FOXA2 in control, patient and CRISPR Patient P1-derived neural progenitors at Day 11 of differentiation. Scale bar = 100 μm. (**B**) Quantification of total number of FOXA2-positive cells and LMX1A/FOXA2 double-positive cells at Day 11 (*n* = 3 for all). (**C**) Representative immunofluorescence images for MAP2 and TH in control, patient and CRISPR Patient P1-derived midbrain dopaminergic (mDA) neurons at Day 65 of differentiation. Scale bar = 150 μm. (**D**) Quantification of total number of MAP2 and TH positive cells, and TH/MAP2 double-positive cells at Day 65 of differentiation (*n* = 3 for all). (**E**) Representative immunofluorescence images for cCASP3 in control, patient and CRISPR Patient P1-derived mDA neurons at Day 65 of differentiation. Scale bar = 150 μm. (**F**) Quantification of total numbers of cCASP3-positive cells and cCASP3/TH double-positive cells at Day 65 of differentiation (*n* = 3 for all). ns = not significant.

CME is an important process in neurogenesis and neurodevelopment, and it is involved in distinct developmental signalling cascades.^37^ We thus sought to investigate the effect of auxilin deficiency and impaired CME on development and maturation of mDA neurons. After 11 days of mDA differentiation, all patient and control lines showed upregulation of midbrain precursor-related genes when compared to iPSCs (Supplementary Fig. 3A). Immunofluorescence analysis for midbrain precursor transcription factor FOXA2 showed comparable levels in both patient and control lines (Fig. 3A and B). However, the amount of LMX1A-positive midbrain precursors was significantly reduced in all patient lines when compared to controls (Fig. 3A and B). At Day 65 of differentiation, control- and patient-derived mDA neurons showed upregulation of midbrain regulated genes compared to iPSCs (Supplementary Fig. 3B) and similar levels of dopaminergic neurons (Fig. 3C and D). To evaluate whether our patient-derived dopaminergic cell model shows early neurodegenerative features at Day 65 of differentiation, we performed cleaved caspase-3 (cCASP3) staining. Both control- and patient-derived neuronal cultures showed similar levels of cCASP3-positive neurons at Day 65 of differentiation (Fig. 3E and F).

**Figure 4:**
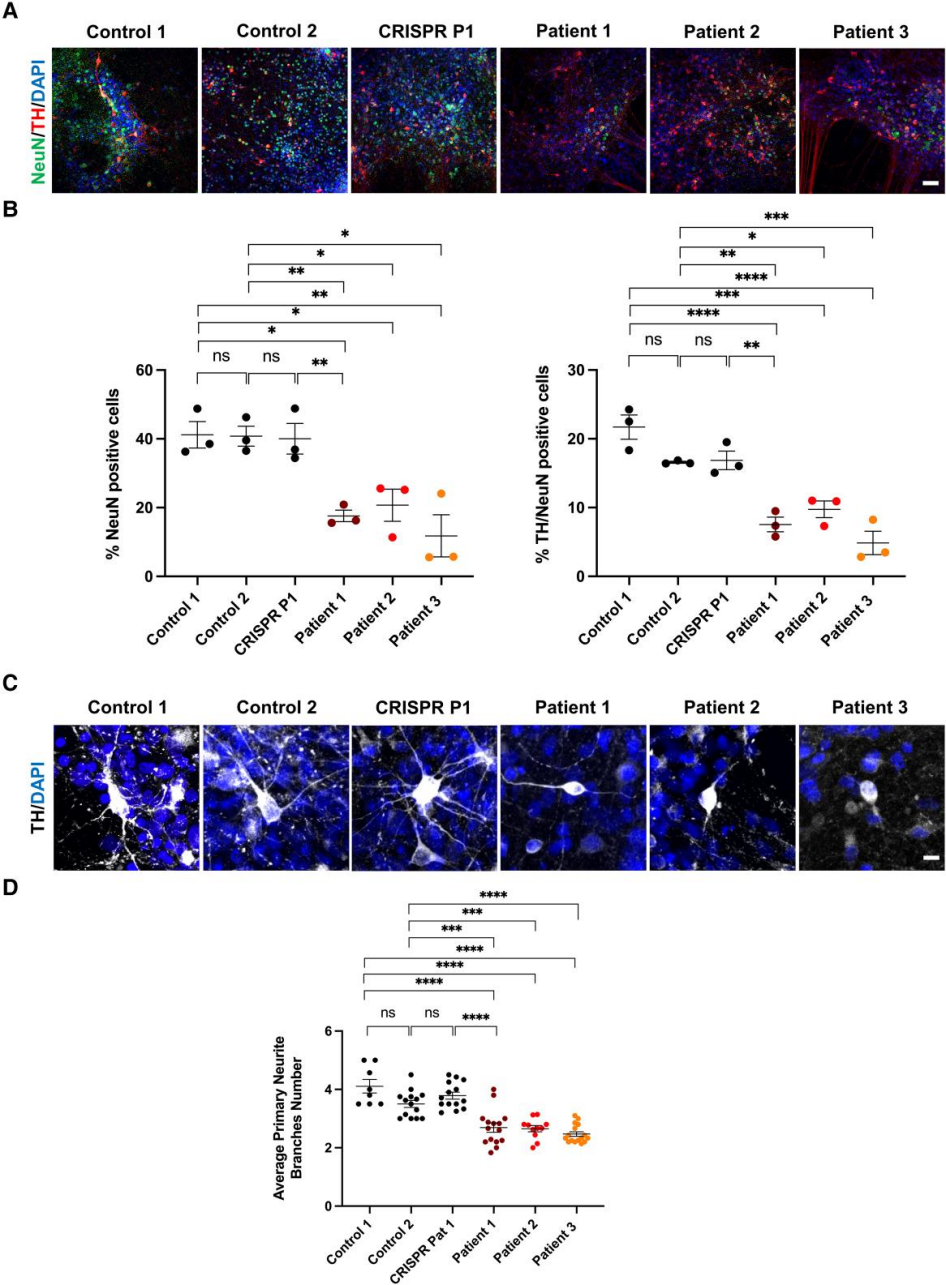
**Patient-derived neurons show defects in neuronal maturation.** (**A**) Representative immunofluorescence images for NeuN and TH in control, patient and CRISPR Patient P1-derived midbrain dopaminergic (mDA) neurons. Scale bar = 150 μm. (**B**) Quantification of NeuN positive and TH/NeuN double-positive cells in control, patient and CRISPR Patient P1-derived mDA neurons at Day 65 of differentiation (*n* = 3 for all). (**C**) Representative immunofluorescence images for control, patient and CRISPR Patient P1-derived mDA neuron branching. Scale bar = 20 μm. (**D**) Quantification of average primary neurite branching in control, patient-and CRISPR Patient P1-derived mDA neurons at Day 65 of differentiation (*n* = 4 for all). ns = not significant.

To assess whether mDA neurons have impaired late-stage neuronal maturation, we performed immunofluorescence analysis of the mature neuronal marker, neuronal nuclear antigen (NeuN). NeuN levels were significantly decreased in all patient mDA lines when compared to controls (Fig. 4A and B). NeuN expression in mDA neurons was generally lower and more variable than MAP2 expression, but comparable to published work.^26^ A study investigating NeuN expression in dopaminergic neurons in the rat substantia nigra has shown that NeuN expression is more variable and a significant proportion of dopaminergic neurons express little or no NeuN and non-dopaminergic neurons generally express higher levels of NeuN.^38^ Though this finding might not directly translate to human iPSC-derived dopaminergic neurons, it does highlight the variability of NeuN expression in mammalian dopaminergic neurons. Finally, we further performed analysis of primary neurite branching and found that all patient mDA lines showed a significant decrease in the number of primary neurites when compared to controls (Fig. 4C and D).

### Disease-specific dysregulated gene expression in key developmental and synaptic pathways

**Figure 5:**
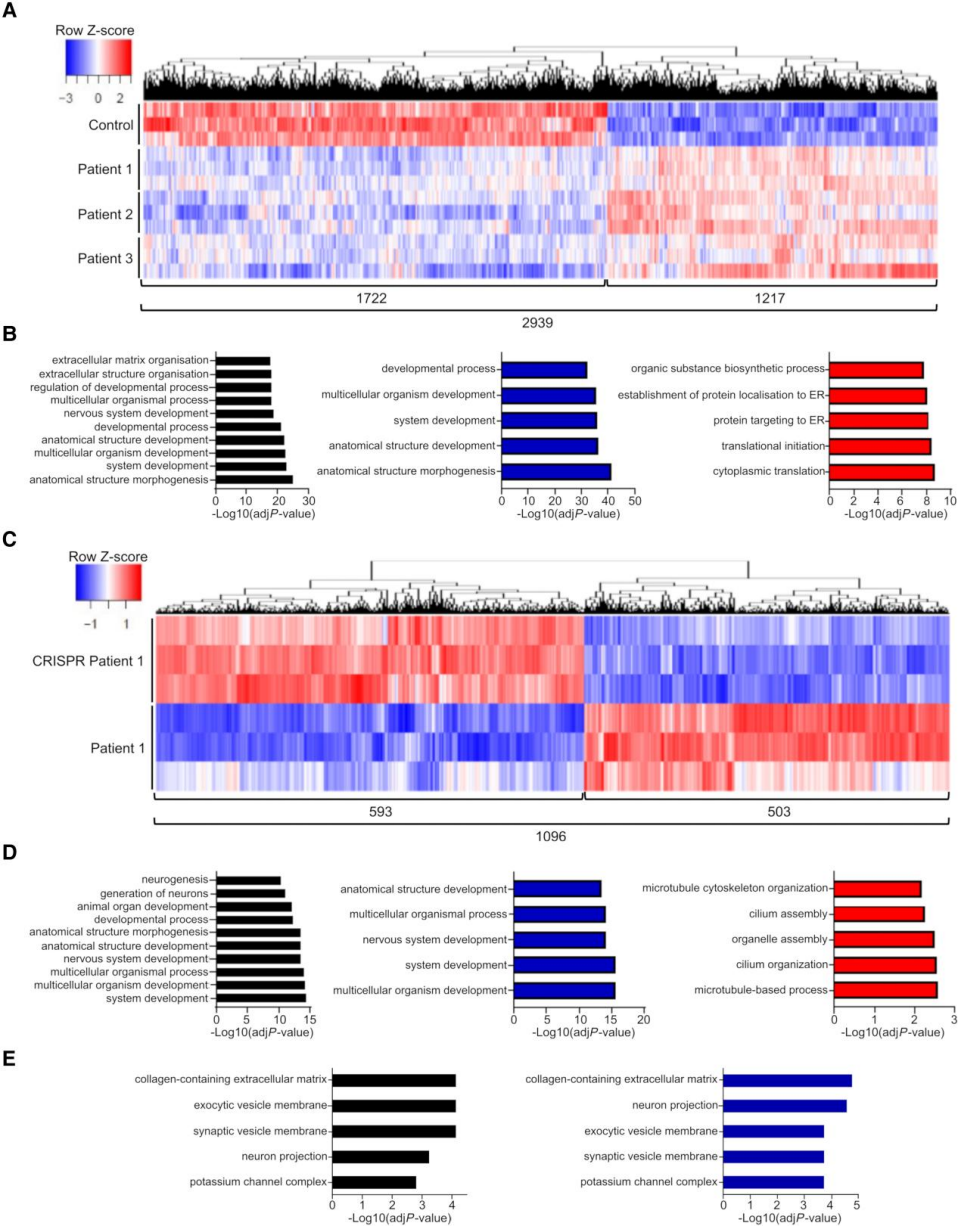
**Bulk RNA sequencing analysis highlights developmental and synaptic vesicle defects in patient-derived neurons.** (**A**) Heat map showing hierarchical clustering of protein-coding differentially expressed genes (DEGs) in all patients compared to Control 1 (*n* = 3). (**B**) Gene Ontology (GO) terms enrichment for biological process of total (black), under-expressed (blue) and over-expressed (red) protein-coding DEGs in patients compared to control. The top 10 or five categories are shown. (**C**) Heat map showing hierarchical clustering of protein-coding DEGs in Patient 1 compared to CRISPR Patient P1 (*n* = 3). (**D**) GO terms enrichment for biological process of total (black), under-expressed (blue) and over-expressed (red) protein-coding DEGs in Patient 1 compared to CRISPR Patient P1. The top 10 or five categories are shown. (**E**) GO terms enrichment for cellular component of total (black) and under-expressed (blue) protein-coding DEGs in Patient 1 compared to CRISPR Patient P1.

Transcriptomic analysis, through bulk RNA sequencing, was undertaken for analysis of protein-coding DEGs. Combined analysis of patients against control neuronal cultures showed 2939 DEGs, of which 58.5% were under-expressed (Fig. 5A). GO annotation revealed a general enrichment in the overall DEGs for genes involved in morphogenesis and developmental processes, including ‘development of the nervous system’ (Fig. 5B, black). Developmental processes were enriched in the under-expressed genes (Fig. 5B, blue), whereas overexpressed DEGs mainly encompassed protein transcription and biosynthetic processes (Fig. 5B, red). To further unravel expressional differences independent of genetic background, we compared Patient 1 DEGs with its isogenic control CRISPR P1 (Fig. 5C–E). A total of 1096 protein-coding DEGs were identified, of which ∼54% were under-represented in the patient neurons (Fig. 5C), with general enrichment in ‘nervous system development’ and ‘neuron generation’ (Fig. 5D, black).

We then analysed downregulated DEGs involved in ‘nervous system development’ and found 61 downregulated genes (12.1% of all downregulated DEGs) (Supplementary Table 3), while in all patients versus Control 1, we found 114 downregulated genes (9.3% of all downregulated DEGs) with an overlap of 21 genes (Supplementary Table 3). In Patient 1 versus CRISPR Patient P1, downregulated DEGs showed a strong enrichment in histones that are involved in ‘Wnt signalling’ (*H2BC8*, *H2BC6*, *H2BC7*, *H2BC5*, *H2BC21*, *H2BC11*, *H2AC6*, *H2BC4*, *H4C5*, *H2AC8*, *H2AJ*, *H2BC12*, *H2BC15*, *H3-3B*). In all patients versus Control 1, a large number of genes (total 50 genes) are involved in ‘extracellular matrix organization’ (*COL2A1*, *COL3A1*, *COL4A1*, *COL4A2*, *COL4A3*, *COL4A4*, *COL4A5*, *COL5A1*, *COL5A2*, *COL6A1*, *COL6A2*, *COL6A3*, *COL9A1*, *COL9A2*, *COL9A3*, *COL6A6*, etc).

Similarly, analysis of downregulated DEGs involved in ‘dopaminergic neurogenesis’ in Patient 1 versus CRISPR Patient P1 revealed seven downregulated genes (*NR4A2*, *NEUROD1*, *LMX1A*, *FOXA2*, *SLC18A2*, *LMX1B*, *DDC)* and in all patients versus Control 1, 11 downregulated genes (*EN1*, *NKX6-1*, *LMX1A*, *LMX1B*, *NEUROD1*, *SHH*, *FOXA2*, *SLC18A2*, *GLI2*, *NR4A2*, *MSX1*). Downregulated DEGs are mainly involved in midbrain floor specification (*LMX1A*, *LMX1B*, *MSX1*) and ventral patterning (*FOXA2*, *GLI*, *SHH*).

**Figure 6:**
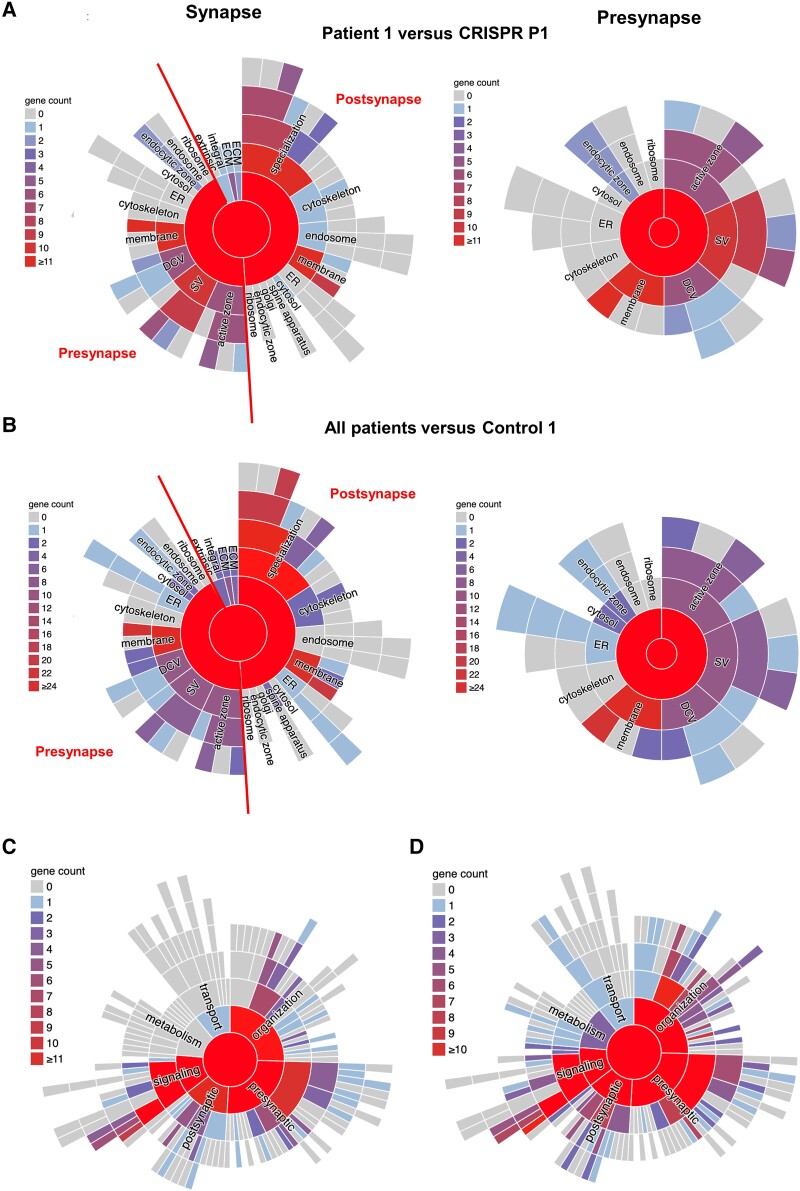
**Synaptic location enrichment analysis reveals structural abnormalities in patient-derived neurons.** (**A**) Pie charts showing synaptic location mapping of under-expressed protein-coding differentially expressed genes (DEGs) in Patient 1 compared to CRISPR Patient P1. *Left*: general synapse; *right*: presynapse zoom-in. (**B**) Pie charts showing synaptic location mapping of under-expressed protein-coding DEGs in all patients compared to Control 1. *Left*: general synapse; *right*: presynapse zoom-in. (**C**) Pie charts showing synaptic function mapping of under-expressed protein-coding DEGs in Patient 1 compared to CRISPR Patient P1. (**D**) Pie charts showing synaptic function mapping of under-expressed protein-coding DEGs in all patients compared to Control 1.

Analysis of GO cellular components in Patient 1 versus CRISPR Patient P1 revealed enrichment in ‘neuron projection’, ‘exocytic vesicle membrane’ and ‘synaptic vesicle membrane’ (Fig. 5E). To further investigate the effect of auxilin deficiency on synaptic homeostasis in *DNAJC6*-related disease, we performed a SynGO analysis of under-expressed coding DEGs (Fig. 6A–C). Analysis of synaptic location showed a strong association with ‘SV’ (synaptic vesicle), in particular ‘SV membrane’ (Fig. 6A and B, left panels and Supplementary Table 4). Further presynaptic locations with high gene counts include ‘presynaptic active zone’, ‘presynaptic endocytic zone’, ‘neuronal dense core vesicles’, ‘presynaptic membrane’ and ‘synaptic cleft’ (Fig. 6A and B, right panels and Supplementary Table 4). Among the downregulated presynaptic genes, we notably found *DNAJC6* involved in ‘presynaptic endocytic zone’ (Supplementary Table 4). We further noticed downregulation of membrane transporters including (i) *SLC17A7*, a sodium-dependent phosphate transporter associated with the membrane of synaptic vesicles and involved in glutamate transport; (ii) *SLC17A6*, a sodium-dependent inorganic phosphate co-transporter involved in neurotransmitter loading into synaptic vesicles; and (iii) *SLC17A8*, a vesicular glutamate transporter. We also found downregulation of neurotransmitter receptors located at the presynaptic membrane such as *GRIK1* (glutamate ionotropic receptor kainate type subunit 1), *GRIK4* (glutamate ionotropic receptor kainate type subunit 4), *GLRA3* (glycine receptor), *GRIN2A* (glutamate ionotropic receptor), *GABRR1* (gamma-aminobutyric acid type A receptor subunit Rho1) and *DRD1* (dopamine receptor D1). Several of these membrane transporters (*SLC17A6*, *SLC18A2*) and receptors (*GABRB2*, *GABRB1*, *GLRA1*, *GLRA2*, *DRD2*) are involved in ‘neuron projection’.

Analysis of synaptic function in Patient 1 versus CRISPR Patient P1 and in all patients versus Control 1 (Fig. 6C and D) further revealed enrichment of genes involved in ‘synaptic signalling’ and ‘synaptic organization’ (Supplementary Table 5). Among the downregulated DEGs we found membrane receptors (*GABRR1*, *DRD1*, *DRD2*, *ADRA2A*, *GRM3*), but also genes associated with cytoskeleton (*TUBB2B*, *TUBA1A)* and extracellular matrix (*TNR*, *NCAM1*). Individual genes are involved in the regulation of neurite outgrowth (*TNR*), axon growth (*NTGN1*) and neuron migration (*GPM6A*, *MDGA1*).

### Proof-of-concept lentiviral gene therapy restores auxilin expression and CME in patient-derived neurons

**Figure 7:**
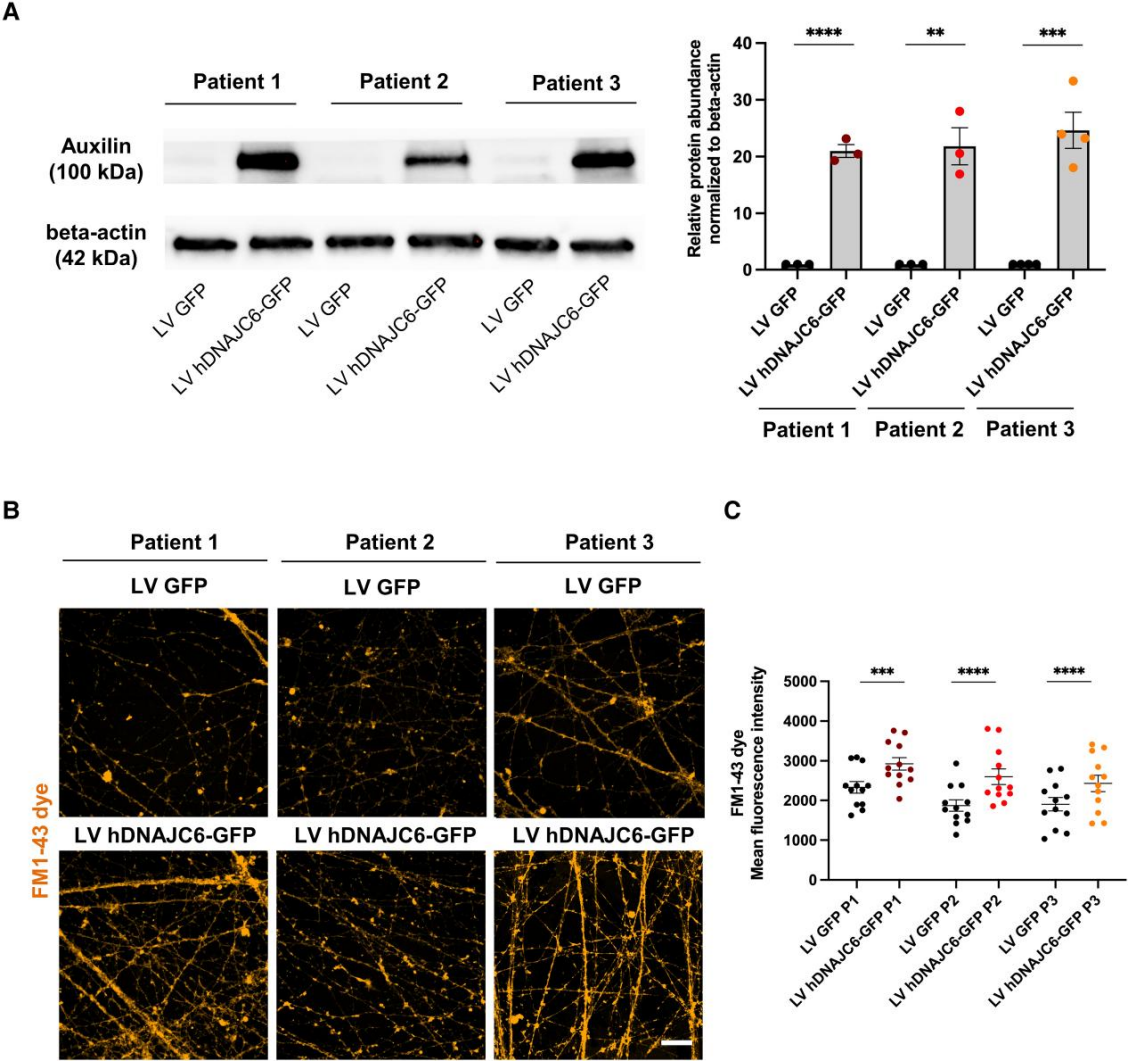
**Lentiviral *DNAJC6* gene transfer restores auxilin and improves CME.** (**A**) *Left:* Representative immunoblot images for auxilin and loading control (β-actin) in patient-derived midbrain dopaminergic (mDA) neurons transfected with LV GFP (lentivirus encoding green fluorescent protein) and LV DNAJC6-GFP (lentivirus encoding DNAJC6-green fluorescent protein) at Day 65 of maturation. *Right*: Quantification relative to the loading control (β-actin) (*n* = 3). (**B**) Representative immunofluorescence images for FM1–43 dye uptake assay in patient-derived mDA neurons transfected with LV GFP and LV DNAJC6-GFP at Day 65 of maturation. Scale bar = 20 μm. (**C**) Quantification of FM1–43 mean fluorescence intensity in patient-derived mDA neurons transfected with LV GFP and LV DNAJC6-GFP.

To develop a precision medicine approach for *DNAJC6* parkinsonism, we sought to evaluate a gene therapy strategy in the neuronal cell model. We generated a lentiviral backbone containing human *DNAJC6* under the transcriptional control of the neuron-specific promotor, human synapsin (hSYN1) (SupplementaryFig. 6A and B). We transduced patient- and control-derived mDA neuronal cultures at Day 24 of differentiation. At Day 65 of differentiation, we evaluated auxilin in *DNAJC6* lentiviral gene-treated cultures and observed rescue of auxilin protein expression in all patient mDA lines (Fig. 7A). We further assessed the effect of lentiviral gene delivery on CME and observed significantly increased FM1–43 dye uptake in treated patient lines (Fig. 7B and C).

## Discussion

Biallelic mutations in *DNAJC6* cause a complex, early-onset movement disorder with additional neurological and neuropsychiatric features.^2-6^ The movement disorder is mainly characterized by progressive childhood-onset parkinsonism-dystonia that responds poorly to standard medications used in the treatment of PD. Symptomatic treatment can alleviate disease manifestations, but the benefit of common antiparkinsonian medications is often limited and accompanied by severe side effects. Patients with juvenile-onset disease also present with other co-morbidities, including neurodevelopmental delay in infancy and childhood, as well as intellectual disability and seizures.^6^ The development of disease-modifying therapies therefore constitutes an unmet clinical need.

The predominant parkinsonian movement disorder phenotype and abnormal DaTScan™ imaging in patients with *DNAJC6* mutations implicate striatonigral pathology; we have thus developed a patient-derived dopaminergic neuronal cell model to study the molecular pathophysiology underlying *DNAJC6* parkinsonism, identifying key defects in synaptic vesicle recycling and neurodevelopment. We have also used this system to provide proof-of-concept for a gene therapy approach to rescue key molecular disease phenotypes, an approach that has significant potential for future clinical translation.

We generated mDA neurons from three patients harbouring different homozygous *DNAJC6* mutations: an early nonsense mutation (c.766C>T) and an early splice-site mutation (c.801-2A>G) both located in the PTEN domain, and a late nonsense mutation (c.2416C>T) located in the clathrin-binding domain. Edvardson *et al.*^2^ have shown that the c.801-2A>G variant results in premature protein truncation with two abnormal cDNA transcripts lacking either a significant part of the PTEN-like domain or the carboxyl-terminal J-domain.^2^ Patient fibroblasts showed markedly reduced *DNAJC6* mRNA levels. As such, all three mutations in this study are predicted to cause nonsense mediated decay, with impaired protein translation, as evidenced by reduced auxilin levels in patient-derived mDA neurons.

In our iPSC-derived neuronal model, we observed disease-specific reduced endocytic reuptake of FM1–43 dye indicating impaired CME in all three patient lines. Efficient synaptic vesicle recycling is also important for timely acidification of synaptic vesicles required for synaptic vesicle loading.^39^ Neurotransmitter loading is driven by vacuolar ATPases (vATPases) located on the synaptic vesicle membrane surface. vATPase activity is inhibited by the clathrin coat and is immediately restored once CCVs become uncoated by auxilin.^40^ Given these findings, it is likely that perturbation of the uncoating process results in dysregulation of synaptic vesicle homeostasis. This was indeed evident in our mDA neuronal model, where synaptic electron microscopy analysis of Day 65 neuronal cultures demonstrated a disease-specific significant reduction of synaptic vesicles at the presynaptic terminal, indicating impaired synaptic vesicle recycling. Transcriptomic analysis further revealed downregulation of transporters (*SLC17A6*, *SLC18A2*) that are responsible for the loading of neurotransmitter into synaptic vesicles. Reduced numbers of presynaptic synaptic vesicle is also in line with data from the R857G auxilin knock-in (KI) mouse mimicking the human R927G mutation located in the terminal J-domain of auxilin^41^; here the authors found reduced numbers of presynaptic synaptic vesicles in the dorsal striatum of 6-month-old KI mice. Reduced synaptic vesicle density has also been observed in other animal models of PD, in particular the synaptojanin *SJ1*^RQ^ KI mouse and *LRRK2* G2019S transgenic mouse.^21,42^ In both mouse models, synaptic vesicle recycling dysfunction was accompanied by axon degeneration and selective dopaminergic neurodegeneration. Decreased loading of dopamine into cycling synaptic vesicles with subsequent accumulation of cytosolic dopamine and disturbance of dopamine homeostasis might, in part, explain the observed selective dopaminergic vulnerability. Reduced synaptic vesicle dopamine loading is also evident in patients, where reduced CSF homovanillic acid (HVA) levels indicate impaired dopamine turnover.^6^

Recently, Wulansari *et al*.^43^ demonstrated dopaminergic neurodegeneration in 80-day-old human midbrain-like organoids (hMLOs) harbouring *DNAJC6* mutations. PD-associated pathological features, such as α-synuclein aggregation and accumulation of intracellular reactive oxygen species, were only evident at Day 130 of differentiation. In our 2D dopaminergic cell model we could not detect any neurodegenerative features at Day 65 on both immunocytochemistry and RNA-seq analysis. It is well acknowledged that iPSC-derived neurons represent fetal human development even at derived neuronal maturity.^44^ The use of later-stage hMLOs or induction of cellular ageing *in vitro* (e.g. progerin-induced ageing and telomere manipulation techniques) might be a future option to overcome this limitation in our study. Indeed, improved derived neuronal maturity may potentially reveal later-onset neurodegenerative phenotypes.^45,46^

Dysfunction and dysregulation of CME machinery has been associated with a wide variety of neurological diseases including epilepsy, Huntington’s disease, PD and Alzheimer’s disease.^47-50^ There is growing evidence that disturbance of CME-associated proteins also causes neurodevelopmental disorders as demonstrated in patients with mutations in *DNM1* and *AP2M1.*^51,52^ CME is involved in a variety of developmental signalling pathways (e.g. RTK, TGF-β/DPP, Hedgehog, Wnt and Notch) and leads to termination or activation of the signal transduction via internalization of distinct transmembrane receptors or intracellular trafficking for endolysosomal recycling.^53^ In neural progenitor cells, CME is important for the distribution of cell fate determinants and ensures balance in proliferation and neurogenesis.^37^ In developing and migrating neurons, CME regulates adhesion/de-adhesion during migration, neuronal polarization and axonal/neurite outgrowth.^37^ In mature neurons, CME is involved in synaptogenesis, transmembrane receptor signalling and dendritic growth arborization and pruning.^37^ In a *Drosophila* model, auxilin was shown to be essential for Notch signalling, a developmental pathway that regulates neural stem cell proliferation, survival, renewal and differentiation.^54,55^

*DNAJC6* mutations thus affect a variety of CME-regulated developmental and synaptic signalling processes. Indeed, transcriptomics analysis of Day 65 neuronal cultures showed perturbation of numerous developmental processes, in particular with regard to CNS development. We found a large number of downregulated genes associated with ‘nervous system development’, particularly genes encoding histones (Patient 1 versus CRISPR Patient P1) that are involved in ‘Wnt signalling’ and ‘extracellular-matrix’-associated genes (all patients versus Control 1). ‘Wnt signalling’, in particular the WNT-LMX1A signalling pathway, is also important in dopaminergic neurogenesis. LMX1A is necessary for the specification of mDA neurons in the midbrain floor plate and its expression is regulated by FOXA2 and WNT1.^56^ In our patient-derived neuronal cultures, we found reduced LMX1A positivity at Day 11 of differentiation, while FOXA2 positive number was comparable to controls, suggesting dysfunctional WNT signalling. Transcriptomic analysis confirmed disturbance of ‘dopaminergic neurogenesis’ with downregulation of genes involved in midbrain floor specification, ventral patterning and WNT signalling.

In Day 65-old mDA neurons, we found further decreased staining of the mature neuronal marker NeuN and reduced primary neurite branching. Neuronal branching is governed by the spatial and temporal expression of neuronal cell surface and signalling molecules, many of which are regulated by CME.^57-59^ Interestingly, transcriptomics analysis of downregulated DEGs involved in ‘neuron projection’ in Patient 1 versus CRISPR Patient P1 showed downregulation of membrane transporters and receptors. SynGO, a synapse-focused analysis of downregulated DEGs in synaptic function revealed a general enrichment in ‘synaptic signalling’ and ‘synaptic organization’ and notably included genes involved in neurite and axon outgrowth, and neuronal migration.

Given the strong enrichment in synaptic vesicle membrane in the GO cellular component analysis of Patient 1 versus CRISPR Patient P1, we specifically explored alterations in under-expressed synaptic genes. Downregulated presynaptic DEGs were strongly linked to ‘SV’, particular ‘SV membrane’, but also to ‘presynaptic active zone’, ‘presynaptic membrane’, ‘neuronal DCV’ and ‘synaptic cleft’. Interestingly, downregulated presynaptic genes included transporters involved in the loading of neurotransmitters into SV vesicles and neurotransmitter receptors located at the presynaptic membrane. Neuronal dense core vesicle (DCV) contain neuropeptides that are important for brain development and synaptic plasticity.^60^ Clathrin has been implicated in the biogenesis of DCV in the trans-Golgi network (TGN) and depletion of clathrin leads to a reduction of DCV.^61^ Depletion of auxilin may therefore also affect the uncoating of other intracellular vesicles such as neuronal DCV. Indeed, in the R857G auxilin KI mouse, auxilin co-localized with the TGN and interacted with a Golgi-resident clathrin-adaptor protein indicating a role of auxilin in the uncoating of TGN-derived CCVs.^41^ Impaired CME thus affects vesicular trafficking to and from several presynaptic compartments (presynaptic membrane, synaptic vesicle , TGN).

In summary, both experimental and transcriptomics analysis demonstrate dysregulation of synaptic vesicle cycling and strongly support a neurodevelopmental phenotype in *DNAJC6* parkinsonism. The impairment of CME by auxilin deficiency is likely to disturb presynaptic membrane homeostasis and the regulation of presynaptic membrane transporters and receptors involved in developmental and synaptic signalling. Many neurodevelopmental processes, such as neurogenesis and neuronal differentiation, neuronal migration and neurite outgrowth, are controlled by ligands and receptors that are regulated by CME. Dysregulation of neurodevelopmental processes are likely to contribute to the early neurodevelopmental features observed in juvenile-onset disease. To further investigate developmental defects caused by auxilin deficiency, future studies in cortical organoid models would also be beneficial.

Gene therapy approaches are rapidly emerging for rare intractable neurogenetic disorders.^62-65^ In this study, we have demonstrated successful restoration of auxilin and CME using a lentivirus approach in our mDA cell model. Whilst not directly translational, this early proof-of-concept will accelerate preclinical work—focused on optimizing promoter, viral vector subtype and intraparenchymal delivery route in animal models—towards this goal.

In conclusion, our humanized *DNAJC6* neuronal cell model not only provides a useful tool for dissecting the molecular pathogenesis of *DNAJC6* parkinsonism, but also serves as a platform for testing novel precision-medicine approaches, which will drive much-needed future translational strategies for this drug-resistant condition.

## Supplementary Material

awae020_Supplementary_Data

## Data Availability

Data that support the findings of this study are available within this article and its Supplementary material. Raw data supporting the findings of this study are available from the corresponding author upon reasonable request.
