# Supplementary material for: Neurodevelopmental and synaptic defects in DNAJC6 parkinsonism, amenable to gene therapy
Source: Brain. 2024 Jan 18;147(6):2023–37. doi: 10.1093/brain/awae020 (PMC11146427; doi:10.1093/brain/awae020)
Supplement: awae020_Supplementary_Data [file awae020_supplementary_data.zip › brain-2023-01299-File010.pdf]

Neurodevelopmental and synaptic defects in *DNAJC6* parkinsonism, amenable to gene therapy

Lucia Abela,[1] Lorita Gianfrancesco,[1] Erica Tagliatti,[2,3] Giada Rossignoli,[1] Katy Barwick,[1] Clara Zourray,[1,2] Kimberley M. Reid,[1] Dimitri Budinger,[1] Joanne Ng,[1,4] John Counsell,[1] Arlo Simpson,[1] Toni S. Pearson,[5,6,7] Simon Edvardson,[8] Orly Elpeleg,[8] Frances M. Brodsky,[9] Gabriele Lignani,[1,2] Serena Barral[1] and Manju A. Kurian[1,10]

Author affiliations:

1 Department of Developmental Neurosciences, Zayed Centre for Research into Rare Disease in Children, UCL Great Ormond Street Institute of Child Health, London, WC1N 1DZ, UK

2 Department of Clinical and Experimental Epilepsy, UCL Queen Square Institute of Neurology, University College London, London, WC1N 3BG, UK

3 Laboratory of Pharmacology and Brain Pathology, Humanitas Clinical and Research Center, Via Manzoni 56, 20089 Milano, Italy

4 Gene Transfer Technology Group, UCL-Institute for Women's Health, London, WC1E 6AU, UK

5 Department of Neurology, Columbia University Irving Medical Center, New York, NY 10032-3784, USA

6 Department of Pediatrics, Nationwide Children's Hospital, Ohio State University, Columbus, OH 43210, USA

7 Department of Neurology, Nationwide Children's Hospital, Ohio State University, Columbus, OH 43210, USA

8 Department of Genetics, Hadassah, Hebrew University Medical Center, 9574869 Jerusalem, Israel

9 Research Department of Structural and Molecular Biology, Division of Biosciences, University College London, London WC1E 6BT, UK

10 Department of Neurology, Great Ormond Street Hospital, London WC1N 3JH, UK

Supplementary Figures:

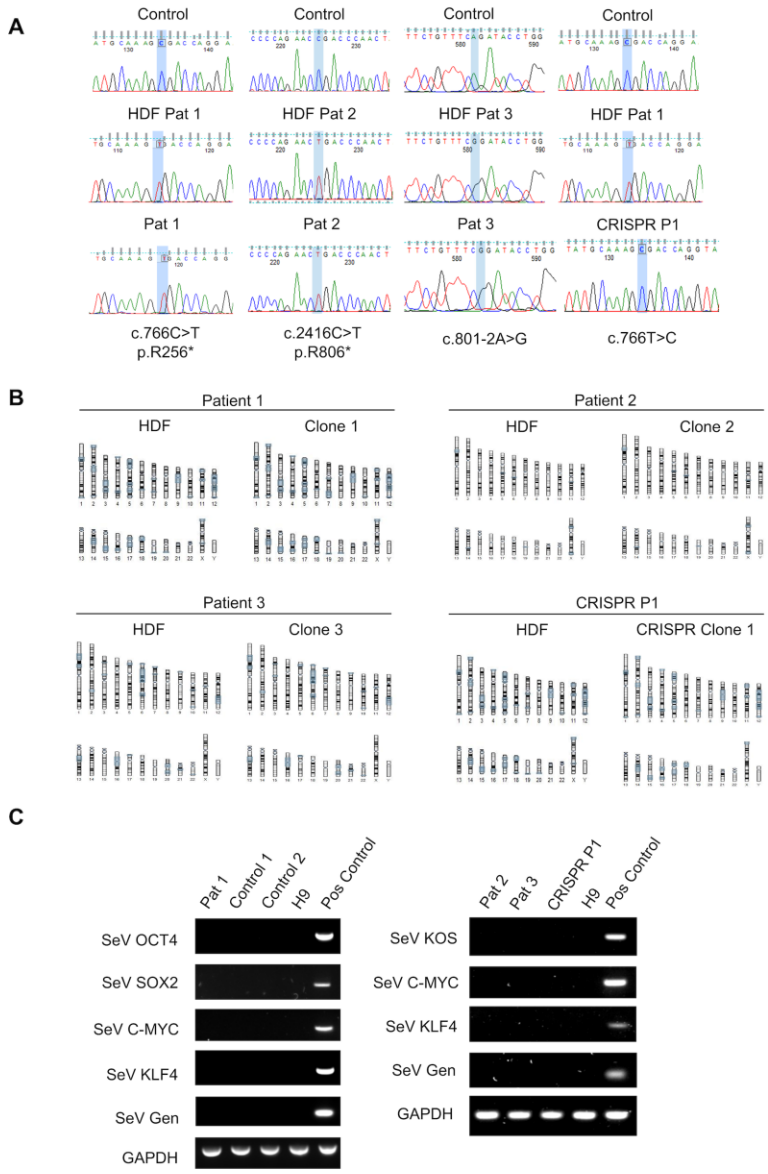

Supplementary Figure 1. Patients-derived iPSC lines retain patient-specific *DNAJC6* mutations and preserve genomic integrity. (A) Sequencing chromatograms of Control and Patients human dermal fibroblasts (HDF) and corresponding iPSC lines demonstrating retention of the corresponding *DNAJC6* mutation after reprogramming. **(B)** Illumina Infinium HumanCytoSNP-12 v2.1 BeadChip array analysis for Patient HDF and corresponding iPSC lines (Patient 1, Patient 2, Patient 3, CRISPR Pat 1). **(C)** RT-PCR for exogenous Sendai Virus genes (SeV) *OCT4, SOX2, KLF4* and *c-MYC* in Patient-derived iPSCs, human Embryonic Stem Cells (H9) and positive control.

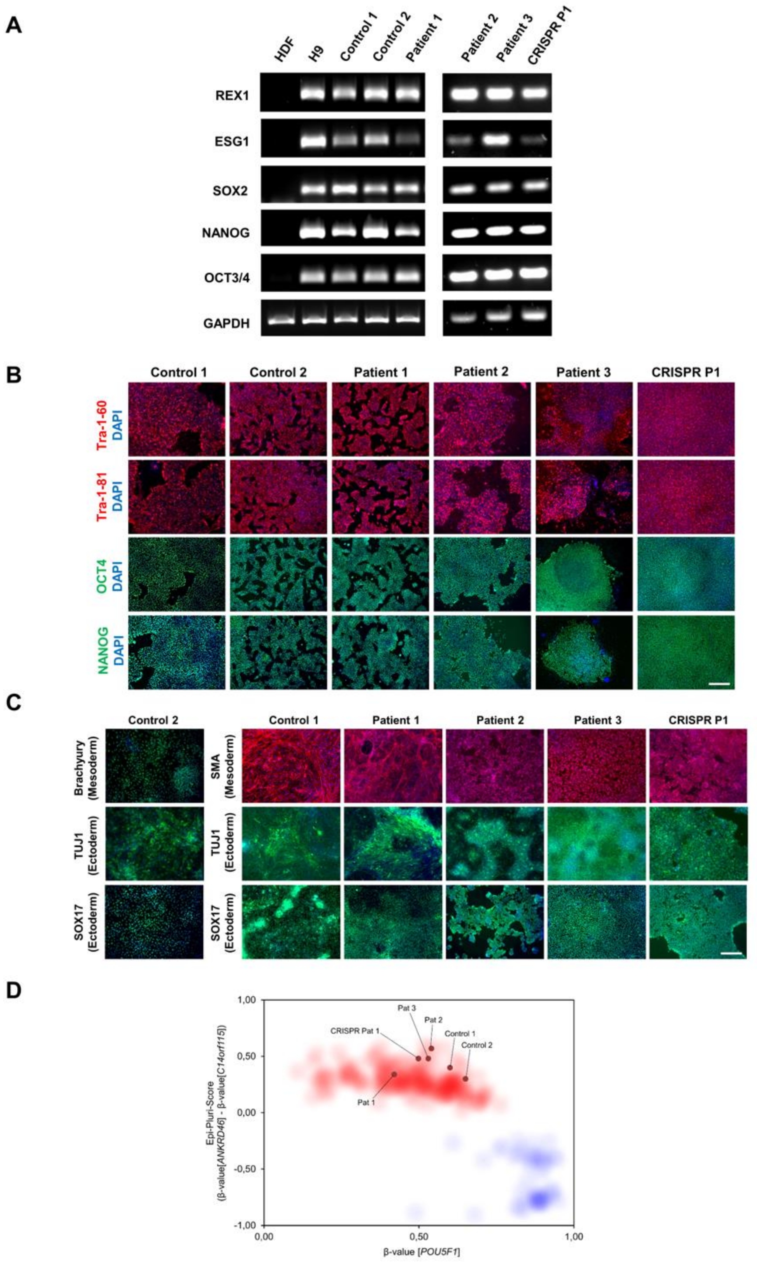

Supplementary Figure 2. Control and patient-derived iPSC lines show true pluripotency characteristics. (A) RT-PCR for the expression of pluripotency genes *REX1*, *ESG1*, *SOX2*, *NANOG* and *OCT3/4* in HDF, human Embryonic Stem Cells (H9), Control and Patient-derived iPSC lines. **(B)** Representative immunofluorescence images for OCT3/4, TRA-1-60, TRA-1-81 and NANOG in Control and Patient-derived iPSC lines. Scale bar = 100 µm. **(C)** *In vitro* spontaneous differentiation assay with representative immunofluorescence images for SOX17 (endoderm), TUJ1 (ectoderm) and SMA/Brachyury (mesoderm) in Control and Patient-derived iPSC lines. Scale bar = 100 µm**. (D)** Epi-Pluri-Score analysis for pluripotency gene expression based on DNA-methylation profiles of 264 pluripotent (Red cloud) and 1,951 non-pluripotent (blue cloud) samples in Control and Patient-derived iPSC lines (Illumina HumanMethylation27 BeadChip platform).

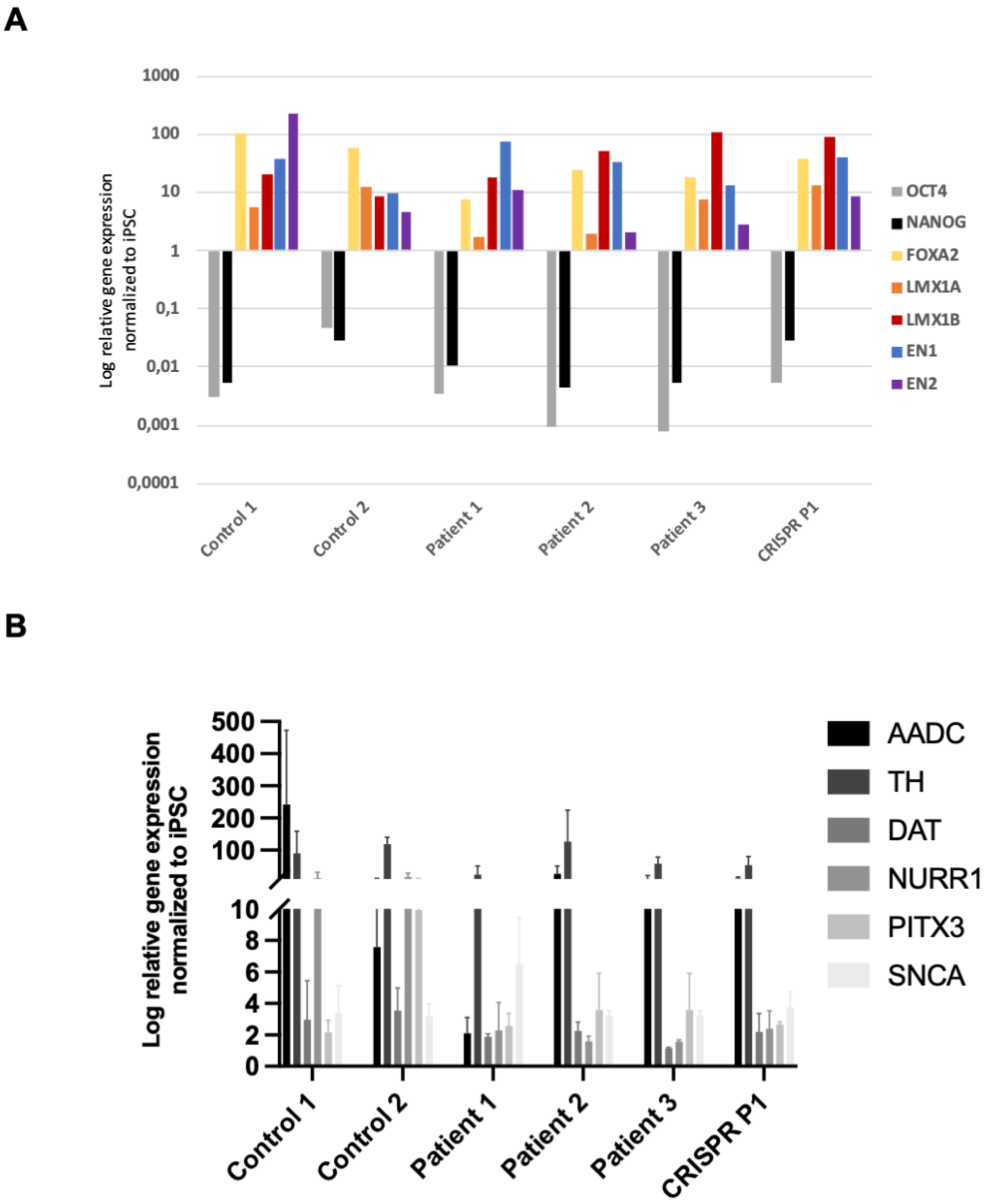

Supplementary Figure 3. Midbrain dopaminergic differentiation and characterization. (A) qRT-PCR of *OCT4* and *NANOG* for pluripotency, and *FOXA2*, *LMX1A*, *LMX1B*, *EN1* and *EN2* for midbrain specification at day 11 of differentiation. mRNA levels are relative to housekeeping gene (*GAPDH*) and normalized to their respective iPSC lines. **(B)** qRT-PCR analyses for *AADC*, *TH*, *DAT*, *NURR1*, *PITX3* and *SNCA* at day 65 of differentiation, relative to the housekeeping gene (*GAPDH*) and normalized to the corresponding iPSC line (n ≥ 3 for all).

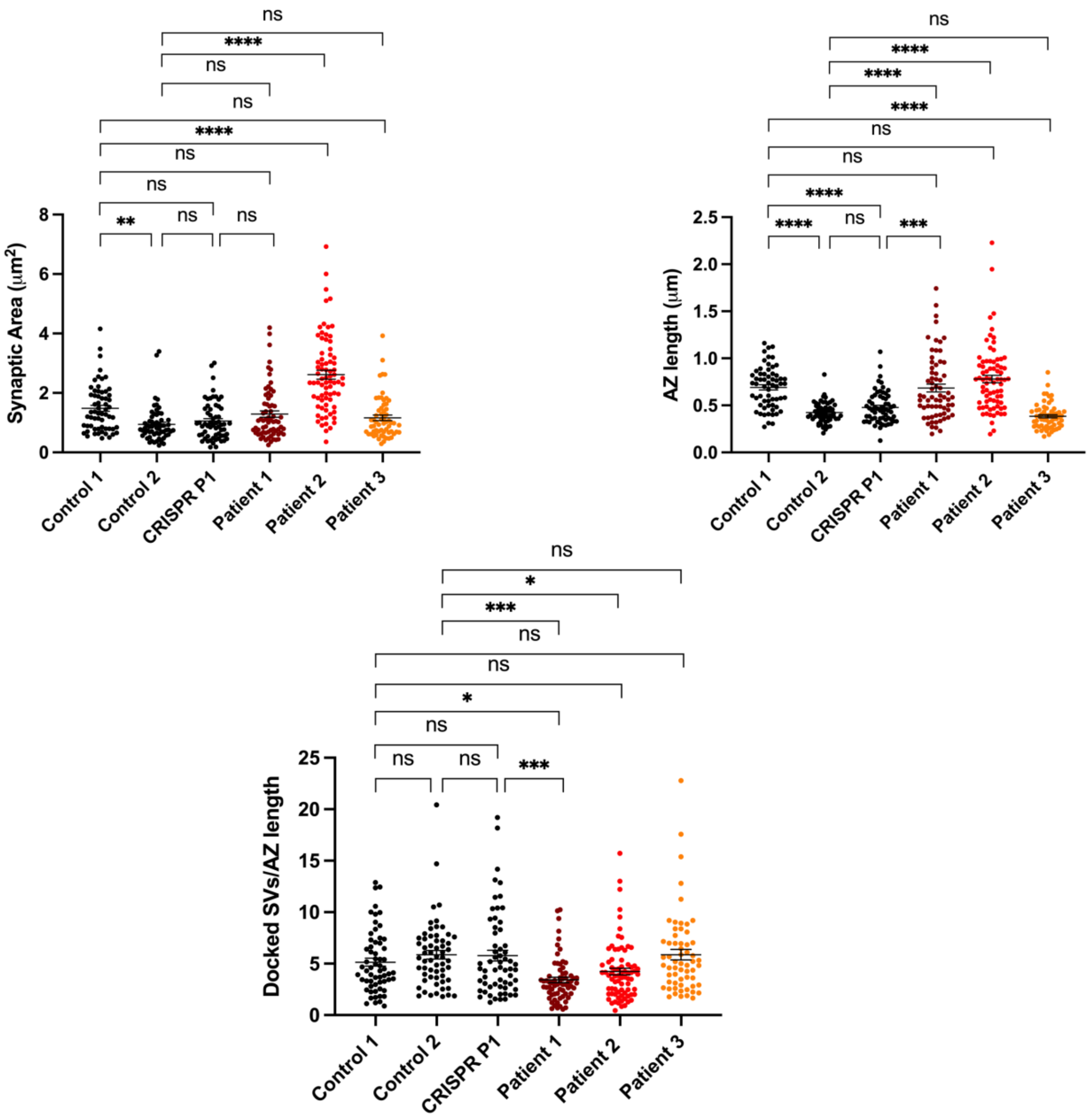

Supplementary Figure 4. Day 65 presynaptic electron microscopy analysis in control and patient lines. Quantification of synaptic area, AZ length and docked SVs/AZ length in Control and Patient-derived mDA neurons at day 65 of differentiation.

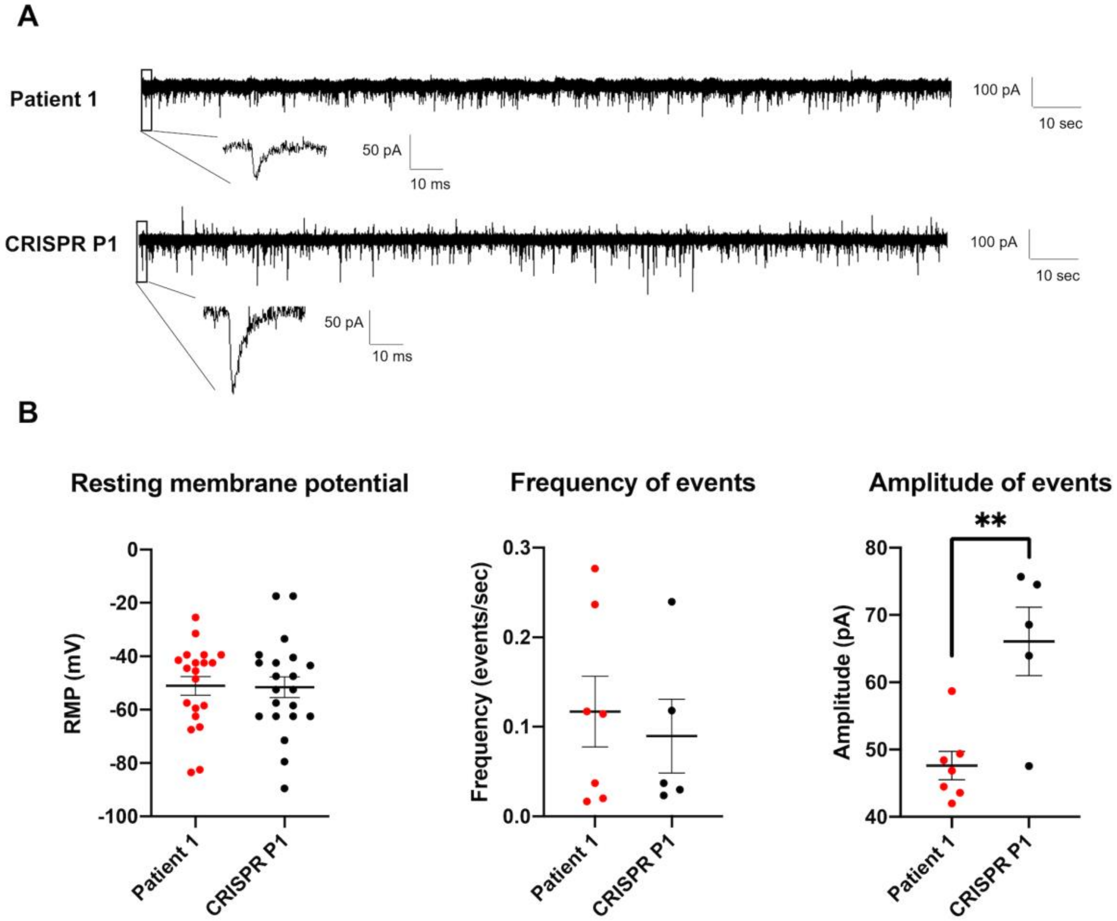

Supplementary Figure 5. Day 70 patch clamp recordings in Patient 1 and its isogenic control CRISPR P1. (A) Representative traces of spontaneous excitatory post-synaptic currents (sEPSCs) recorded from Patient 1 and CRISPR P1-derived dopaminergic neurons at day 70 of differentiation. **(B)** Quantification of resting membrane potential (RPM), frequency of events and amplitude of events in Patient 1 and CRISPR P1-derived dopaminergic neurons.

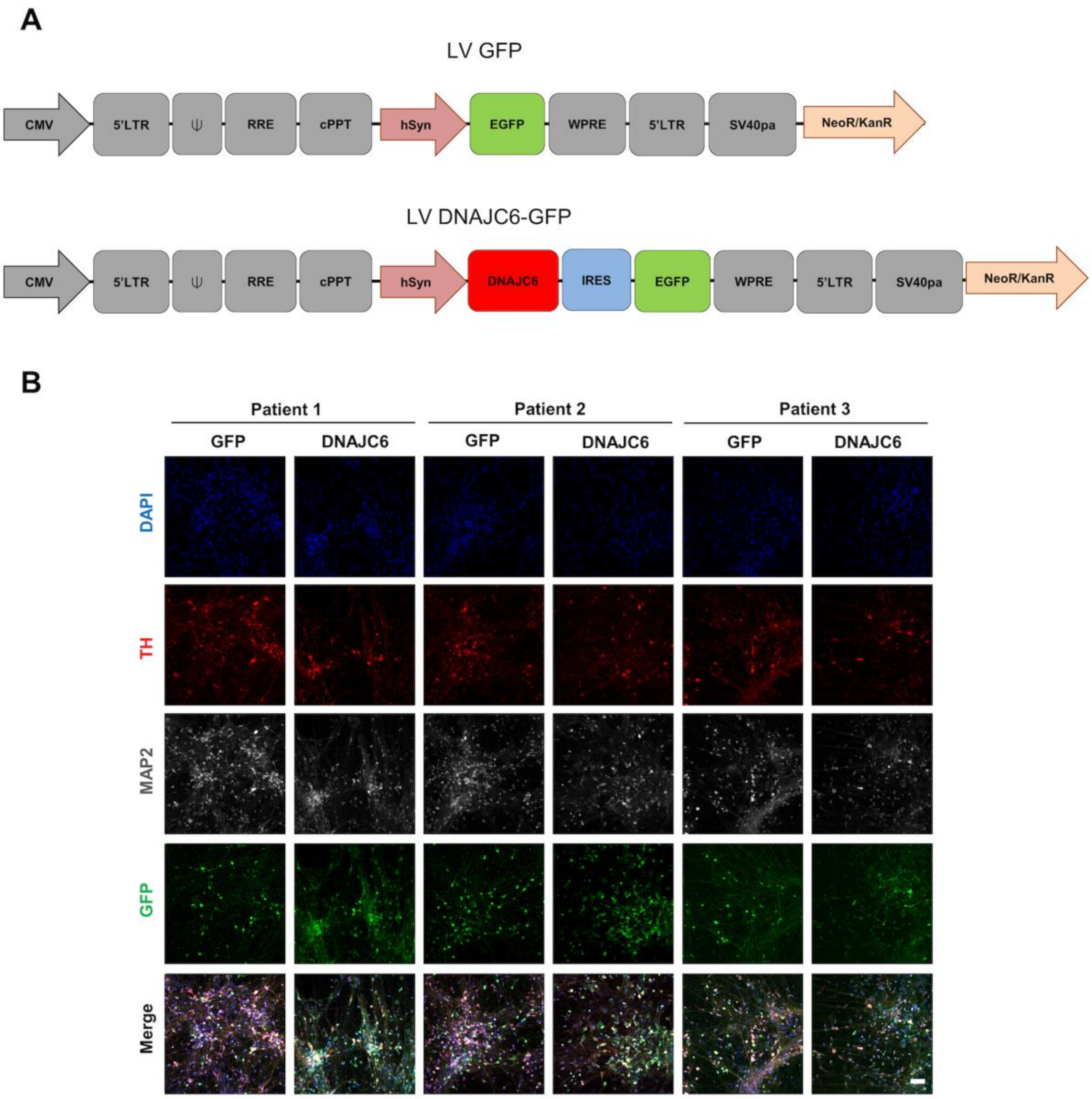

Supplementary Figure 6. Generation of a lentiviral vector for *in vitro DNAJC6* gene transfer. (A) Schematic representation of a lentiviral vector containing an expression cassette with human synapsin promoter (hSYN1) controlling human *DNAJC6* gene (h*DNAJC6*) linked by Internal Ribosome Entry Site sequence (IRES) to GFP gene. **(B)** Representative immunofluorescence images for MAP2, TH and GFP at day 65 of maturation in patient-derived mDA neurons transfected with LV GFP and LV DNAJC6-GFP at day 24 of differentiation. Scale bar 150 μm.

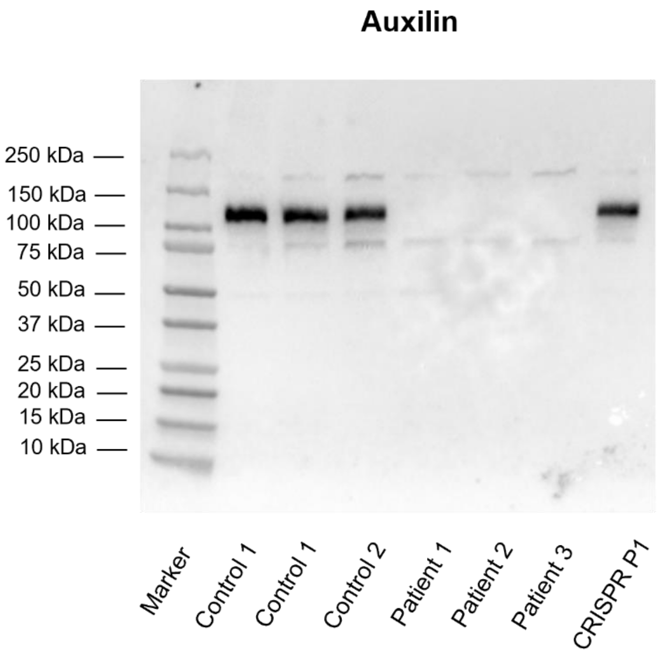

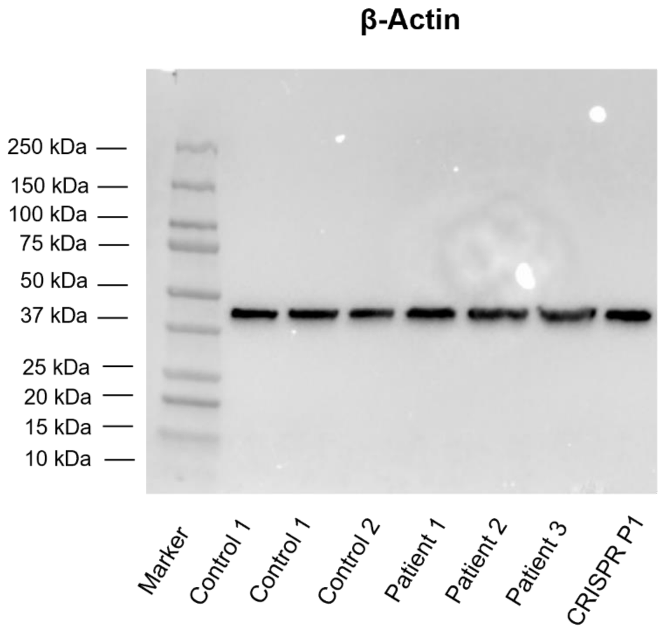

Supplementary Blots. Full length western blots from Figure 2A. Representative immunoblot for auxilin and loading control (beta-actin) from patient-derived neurons at day 65 of differentiation.

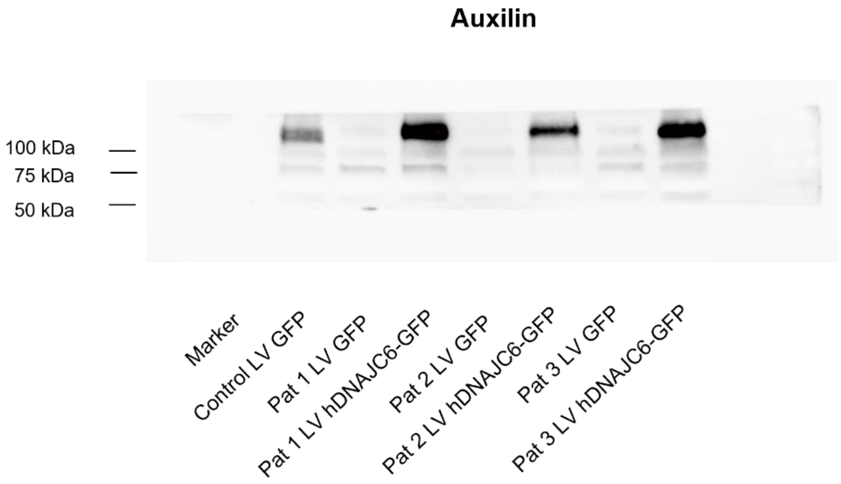

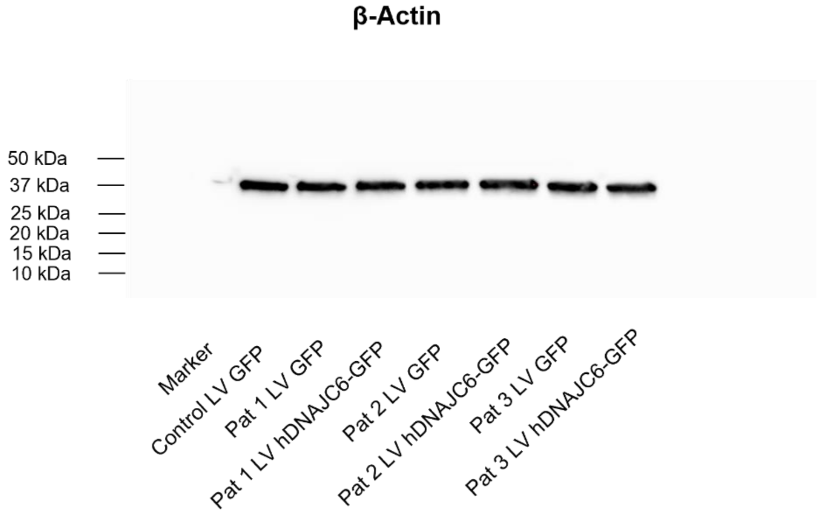

Supplementary Blots. Full length western blots from Figure 7A. Representative immunoblot for auxilin and loading control (beta-actin) from Lentivirus-transfected patient-derived neurons at day 65 of differentiation.

Supplementary Tables:

Name	Use, concentration	Source, catalogue number
beta-actin	IB 1:5000	Sigma-Aldrich, A1978
Auxilin	IB 1:3000	Gift Prof. Lois E. Greene
Brachyury	ICC 1:50	R&D Systems, AF2085
FOXA2	ICC 1:500	BD Pharmigen, 561580
GFP	ICC 1:500	Invitrogen, A6455
LMX1A	ICC 1:500	Millipore, AB10533
MAP2	ICC 1:400	Sigma-Aldrich, M9942
NANOG	ICC 1:500	Millipore, MABD24
NeuN	ICC 1:100	Millipore, MAB377
OCT3/4	ICC 1:50	Santa Cruz Biotech, sc-5279
SMA (alpha smooth muscle actin)	ICC 1:100	Abcam, ab32575
SOX17	ICC 1:400	R&D Systems, AF1924
TH	ICC 1:400	Aves Labs, TYH
TRA-1-60	ICC 1:200	Santa Cruz Biotech, sc21705
TRA-1-81	ICC 1:200	Millipore, MAB4381
TUJ1	ICC 1:400	Biolegend, MMS-435P

Supplementary Table 1. List of antibodies. IB: immunoblotting; ICC: immunohistochemistry

Name	Primer forward sequence	Primer reverse sequence	Use
c-MYC	GCGTCCTGGGAAGGGAGATCCGGAGC	TTGAGGGGCATCGTCGCGGGAGGCTG	RT-PCR
EN1	CGTGGCTTACTCCCCATTTA	TCTCGCTGTCTCTCCCTCTC	qRT-PCR
EN2	CCTCCTGCTCCTCCTTTCTT	GACGCAGACGATGTATGCAC	qRT-PCR
ESG1	ATATCCCGCCGTGGGTGAAAGTTC	ACTCAGCCATGGACTGGAGCATCC	RT-PCR
FOXA2	CCGTTCTCCATCAACAACCT	GGGGTAGTGCATCACCTGTT	qRT-PCR
GAPDH	ATCCCATCACCATCTTCCAG	CCATCACGCCACAGTTTCC	RT-CPR
	TTGAGGTCAATGAAGGGGTC	GAAGGTGAAGGTCGGAGTCA	qRT-PCR
LMX1A	CGCATCGTTTCTTCTCCTCT	CAGACAGACTTGGGGCTCAC	qRT-PCR
LMX1B	CTTAACCAGCCTCAGCGACT	TCAGGAGGCGAAGTAGGAAC	qRT-PCR
NANOG	CAGCCCCGATTCTTCCAGTCCC	CGGAAGATTCCCAGTCGGGTTCACC	RT-CPR
	TTGGGACTGGTGGAAGAATC	GATTTGTGGGCCTGAAGAAA	qRT-PCR
NURR1	TCGACATTTCTGCCTTCTCCTG	GGTTCCTTGAGCCCGTGTCT	qRT-PCR
OCT3/4	CGAAACCCACACTGCAGCAG	CCTGGCACAAACTCCAGGTTT	RT-CPR
	TCTCCAGGTTGCCTCTCACT	GTGGAGGAAGCTGACAACAA	qRT-PCR
PITX3	GAGCTAGAGGCGACCTTCC	CCGGTTCTTGAACCACACCC	qRT-PCR
REX1	CAGATCCTAAACAGCTCGCAGAAT	GCGTACGCAAATTAAAGTCCAGA	RT-PCR
SeV c-MYC	TAACTGACTAGCAGGCTTGTCG	TCCACATACAGTCCTGGATGATGATG	RT-PCR
SeV KLF4	TTCCTGCATGCCAGAGGAGCCC	AATGTATCGAAGGTGCTCAA	RT-PCR
SeV SOX2	ATGCACCGCTACGACGTGAGCGC	AATGTATCGAAGGTGCTCAA	RT-PCR
SeV OCT4	CCCGAAAGAGAAAGCGAACCAG	AATGTATCGAAGGTGCTCAA	RT-PCR
SNCA	GGAGTGGCCATTCGACGAC	CCTGCTGCTTCTGCCACAC	qRT-PCR
SOX2	GGGAAATGGGAGGGGTGCAAAAGAGG	TTGCGTGAGTGTGGATGGGATTGGTG	RT-PCR
TH	CGGGCTTCTCGGACCAGGTGTA	CTCCTCGGCGGTGTACTCCACA	qRT-PCR
WPRE	GTCCTTTCCATGGCTGCTC	CCGAAGGGACGTAGCAGA	qRT-PCR

Supplementary Table 2. List of primers for RT-PCR and qRT-PCR

	CLATHRIN-MEDIATED ENDOCYTOSIS	DOPAMINERGIC NEUROGENESIS	NERVOUS SYSTEM DEVELOPMENT
**PATIENT 1 versus CRISPR P1**			
**Downregulated genes (total 593 DEGs)**	*AMPH, DNAJC6, TACR1, TF*	*DDC, FOXA2, LMX1A, LMX1B, NEUROD1, NR4A2, SLC18A2*	*ACVR1C, ADGRG6, PAX6, CACNA1G, CFC1, CNTN2, COL2A1, COL4A2, DPYSL5, DPYSL3, DSCAML1, EBF1, EFNA1, EFNA3, EOMES, EPHA5, EPHA6, FOXA2, FOXP1, GFRA2, GFRA3,* ***H2AC6, H2AC8, H2AJ, H2BC4, H2BC5, H2BC6, H2BC7, H2BC8, H2BC11, H2BC12, H2BC15, H2BC21,*** *H3-3B,* ***H4C5,*** *HIF3A, HEY2, MAPK13, MYOG, NEUROD1, NOG, NRP2, LHX9, PKP1, POU3F2,* ***PSMB8,*** *MEIS1, RND1, SALL4, SCD5, SCN7A, SEMA3E, SLIT1, SRGAP2, TRPC5, TUBA1A, TUBB2B, TUBB4A, RGMA, VAV3, ZIC3*
**Upregulated genes (total 503 DEGs)**	*REPS1, SLC18A3, STAM2*	-	*COL9A2, CNTN6, CHL1, CXCL12, EFNA5, FGF10, ISL1, LHX2, MYH14, PTPRC*
**ALL PATIENTS versus CONTROL 1**			
**Downregulated genes (total 1722 DEGs)**	*BTC, DAB2, DNAJC6,* ***FZD4,*** *ITSN1, LDLRAP1, LRP2, NECAP2, TACR1, STON1, STON2,* ***WNT5A***	*EN1, FOXA2, GLI2, LMX1A, LMX1B, MSX1, NEUROD1, NKX6-1, NR4A2, SHH, SLC18A2*	*ADGRG6, ADGRV1, AGRN, AJUBA,* ***AKT2,*** *ALCAM, ANK1, ARHGEF28, BOC, EBF1, CACNA1C, CACNA1H, CACNG4, CD36, CDK2, CFC1, COL2A1, COL3A1, COL4A1, COL4A2, COL4A5, COL5A1, COL4A4, COL5A2, COL5A3, COL6A2,* ***COL6A3,*** *COL9A1, COL9A3, CNTN6, CXCL12, DOCK1, DSP, EFNA4, EFNB2, EFNB3, EPHA4, EPHA5, EPHA6, EPHA7, EPHB4, ERBB2, FOXA1, FOXA2, FOXC1, FOXH1, FOXP1, FURIN,* ***H2AX,*** *HES1, HEY2, HNF4G, H4C8, ITGA2, ITGA5, ITSN1, KLF4, KLF5, KMTD2, LAMA2, LAMB1, LAMC1, LEF1, LFNG, MAML2, NCAM1, NEUROD1, NTN1, NTN3, MAML1, MAMLD1, MMP2, MYO10, PBX1, PAX6, PCK1, PLXNB1, PMP22, POU3F2, PSMB8, PTGDS, NKX6-1, MEIS1, MSI1, RGMA, RUNX1,* ***H3C6,*** *RARG, SCN7A, SALL1, SALL4, SEMA5A, SHH,* ***SOX9,*** *SLIT1, SLIT2, SLIT3, SMAD3, SRF, TEAD2, TCF3, TCF4,* ***TCF7,*** *TLN1, SPTB, TRPC4, TRPC5, TRPC6, TUBB6, VAV3, WWTR1, YAP1, ZIC3, ZFPM2*
**Upregulated genes (total 1217 DEGs)**	*ARPC3, CD3G, COPS3, NECAP1, SH3GL3, SLC18A3, TGFA,* ***UBA52,*** *VAMP2,*	*CDKN1C, FGF8, TH*	*ARPC3, CACNG2, CACNG3, CCND3, CDKN1A, CEBPA, CHL1, CNOT9, DHH, DLL3, DSC1, EFNA5, EPHA2, GCK, GRB10, HMGCR, IAPP, ISL1, KRT10, KRT18, LHX2, LIN28A, LPL, MAPK8, MED8, MED10, MET, NGEF, NRTN, NR5A1, PLXNC1, POLR2K, POU3F1, PRSS8,* ***PSMB1, PSMC4, PSMD8, PSMD10,*** *RARB, RPLP2, RPL5, RPL8, RPL9, RPL18, RPL18A, RPL21, RPL22L1, RPL27, RPL31, RPL32, RPL34, RPL35, RPL38, RPL39, RPL39L, RPL41, RPS8, RPS13, RPS16, RPS18, RPS21, RPS29, RBPJ, RPL24, RPS11, RPS27, RPS55, RPS4X, SEMA7A, TCHH, TUBA4A,* ***UBA52,*** *UPF3B*

Supplementary Table 3. Overview on phenotype-associated significantly under- and overexpressed genes. Genes involved in clathrin-mediated endocytosis (146 genes), dopaminergic neurogenesis (30 genes) and nervous system development (1169 genes) in Patient 1 versus CRISPR P1 and all Patients versus Control 1, respectively. Genes highlighted in red are shared between the two groups. Genes in bold are

associated with the Wnt signalling pathway (“signalling by Wnt”). Genes associated with the mentioned GO terms were extracted from Pathcards (https://pathcards.genecards.org/).

GO TERM<br>HIERARCHICAL STRUCTURE	GENES
**PATIENT 1 versus CRISPR P1**	
**Presynapse (total 32 genes)**	*ADRA2A, AMPH, BDNF, DNAJC6, DRD2, ERBB4, GABRR1, GABRB2, GLRA1, GPM6A, GRIK1, GRM3, GRM7, GRM8, GRP, IGF1, KCNA4, KCNJ11, NTNG1, PDYN, PLAT, RPH3A, SEPTIN5, SLC17A7, SLC18A2, TMEM163, SLC17A6, SLC17A8, SV2B, SYNPR, SYT7, WLS*
**Presynaptic active zone**<br>**Integral component of presynaptic active zone**	*ADRA2A, GABRB2, GPM6A, GRM7, GRM8*
**Presynaptic endocytic zone**	*AMPH, DNAJC6*
**Synaptic vesicle**<br>**Synaptic vesicle membrane**<br>**Integral component of synaptic vesicle membrane**	*AMPH, PDYN, RPH3A, SLC17A6, SLC17A7, SLC17A8, SLC18A2, SV2B, SYNPR, TMEM163*
**Neuronal dense core vesicles**	*BDNF, GRP, IGF1, PDYN, PLAT, SLC18A2*
**Presynaptic membrane**<br>**Integral component of presynaptic membrane**	*DRD2, ERBB4, GABRR1, GLRA1, GRIK1, GRM3, GRM7, KCNA4, KCNJ11, SYT7, WLS*
**Synaptic cleft**	*CBLN1, C1QL3, C1QL1, LAMA4, TNR*
**Postsynapse (total 24 genes)**	*ACTN2, ADRA2A, ALDOC, ARC, CACNG5, CACNG7, CNTN2, CHRND, DNAJB1, DRD2, ERBB4, GABRB2, GLRA1, GRM1, GRM3, IL1RAPL1, KCNJ2, KCNA4, NRP2, NTRK3, NRGN, PLAT, SLC18A, WLS*
**Postsynaptic specialisation**	*ACTN2, ADRA2A, ARC, CACNG7, CHRND, DNAJB1, ERBB4, GABRB2 GLRA1, GRM1, IL1RAPL1*
**Postsynaptic membrane**	*ADRA2A, CACNG5, CNTN2 DRD2, GRM1, GRM3, KCNA4, KCNJ2, NRGN, NRP2, NTRK3*
**ALL PATIENTS versus CONTROL 1**	
**Presynapse (total 58 genes)**	*ADD3, ADRA1A, ADRA2A, BDNF, CACNA1C, CACNA2D1, CACNA1H, CALCRL, CADPS2, CDH10, CHRNA6, CNTN6, DNAJC6, DISC1, DOCK1, DRD1, EFNB2, EFNB3, EPHA4, ERBB2, ERBB4, GABRB2, GABRR1, GPC4, GPER1, GRIN3B, GRM8, GRIK1, GRIK4, GLRA3, GRIN2A, GRM3, HTR2A, IGF1, ITPR1, ITSN1, ITGA2, LPAR1, LRRK2, OPRD1, PLAT, PENK, PTN, NCAM1, NPY1R, NTNG1, RGS9, RPH3A, SEPTIN5, SLC6A9, SLC17A6, SLC18A2, STON2, SV2B, SYDE1, TMEM163, TRIM9, WLS*
**Presynaptic active zone**	*ADRA2A, CACNA2D1, CACNA1H, CDH10, GABRB2, GRIN3B, GRM8, NTNG1, SYDE1*
**Presynaptic endocytic zone**	*DNAJC6, ITSN1*
**Synaptic vesicle**<br>**Synaptic vesicle membrane**<br>**Integral component of synaptic vesicle membrane**	*OPRD1, PENK, RPH3A, SLC6A9, SLC17A6, SLC18A2, SV2B, TMEM163, TRIM9*
**Neuronal dense core vesicles**	*BDNF, CACNA2D1, CALCRL, IGF1, NPY1R, OPRD1, PENK, PLAT, SLC18A2*
**Presynaptic membrane**<br>**Integral component of presynaptic membrane**	*ADRA1A, CACNA1C , DRD1, EFNB2, EFNB3, EPHA4, ERBB2, ERBB4, GABRR, GPER1, GLRA3, GRIK1, GRIK4, GRIN2A, HTR2A, KCNC3, LPAR1, NCAM1, NPY1R, OPRD1, SLC6A1, WLS*
**Synaptic cleft**	*AGRN, SPARCL1*
**Postsynapse (total 74 genes)**	*ADRA1A, ADRA2A, APLP2, ADD3, CACNA1C, CACNA1H, CACNA2D1, CACNG4, CACNG5, CALD1, CDH10, CNIH2, CHRNA6, CRTC1, CSPG5, CTTNBP2, CYFIP1, DISC1, DLG5, DMD, DOCK10, DRD1, EPHA4, EPHA7, EFNB2, EFNB3, ERBB4, FLNA, GABRA1, GABRB2, GLRA3, GPER1, GRIN2A,*

	GRIK4, GRIN3B, GRM1, GRM3, HTR2A, ITGA5, ITGA8, ITGB4, ITPR1, ITSN1, KCNJ2, LPAR1, LRRC4, LRRTM3, LYN, MAGI2, MPDZ, NCAM1, NECTIN3, NETO1, NSMF, NTRK2, NTRK3, OPRD1, PLAT, PTN, PTPRO, PTPRZ1, RGS9, ROR2, SIPA1L1, SLC6A9, SLC18A2, SLC30A1, SNAP23, SPTB, SYNPO, TACC3, TANC1, TENM2, WLS
**Postsynaptic specialisation**	*ADRA2A, CACNG4, CDH10, CNIH2, CRTC1, DISC1, DLG5, DMD, EFNB2, EFNB3, EPHA7, ERBB4, GABRA1, GABRB2, GLRA3, GRIN2A, GRIN3B, GRM1, ITGA8, LYN, LRRC4, LRRTM3, MAGI2, MPDZ, NECTIN3, NETO1, OPRD1, PTPRO, PTPRZ1, RGS9, SLC6A9, SLC30A1, TACC3, TANC1*
**Postsynaptic membrane**	*ADRA1A, ADRA2A, CACNA1C, CACNA1H, CACNA2D1, CACNG5, CHRNA6, CSPG5, DRD1, EPHA4, GPER1, GRIK4, GRM1, GRM3, HTR2A, ITGA5, ITGB4, KCNJ2, LPAR1, MAGI2, NCAM1, NTRK2, NTRK3, SLC6A9, TENM2*

Supplementary Table 4. Overview on synaptic phenotype-associated significantly underexpressed genes. Synaptic GO terms (synaptic location) in hierarchical structure with associated significantly underexpressed genes in Patient 1 vs CRISPR P1 and all Patients vs Control 1, respectively.

GO TERM HIERARCHICAL STRUCTURE	GENES
**PATIENT 1 versus CRISPR P1**	
**Presynapse (total 19 genes)**	*AMPH, CALB2, DNAJC6, DRD2, GABRR1, GABRB2, GLRA1, GRIK1, KCNA4, KCNJ11, RPH3A, SEPTIN5, SLC17A6, SLC17A7, SLC17A8, SLC18A2, SV2B, SYT7, TMEM163*
**Regulation of presynaptic membrane potential**	*GABRR1, GABRB2, GLRA1, GRIK1, KCNA4, KCNJ11*
**Synaptic vesicle cycle**	*AMPH, DNAJC6, RPH3A, SEPTIN, SV2B, SYT7, SLC17A6, SLC17A7, SLC17A8, SLC18A2, TMEM163*
**Postsynapse (total 12 genes)**	*ARC, CACNG5, CACNG7, C1QL3, CHRND, ERBB4, GABRB2, GLRA1, GLRA2, GRIK1, GRM1, SYT6*
**Synaptic signalling (total 24 genes)**	*ADRA2A, ARC, BDNF, CBLN2, CCK, CDH11, CHRNA5, DRD2, GABRR1, GRM3, GRM7, GRM8, IGF1, IL1RAPL1, NRGN, NRN1, NTNG1, PLAT, SYNPR, TENM1, TNR, TUBB2B, UBE3A, WLS*
**Transsynaptic signalling**	*ADRA2A, ARC, BDNF, CBLN2, CDH11, CHRNA5, DRD2, GABRR1, GRM3, GRM7, GRM8, IGF1, L1RAPL1, NRN1, NRGN, NTNG1, PLAT, SYNPR, TENM1, TNR, TUBB2B, UBE3A, WLS*
**Chemical synaptic transmission**	*ARC, CBLN2, CDH11, GABRR1, NTNG1, SYNPR, TUBB2B, TNR, UBE3A*
**Synapse organization (total 15 genes)**	*ERBB4, CBLN1, CBLN2, C1QL3, C1QL1, GPM6A, IL1RAPL1, MDGA1, NRP2, NTRK3, NTNG1, SEMA3F, TENM1, TUBA1A, TUBB*
**ALL PATIENTS versus CONTROL 1**	
**Presynapse (total 25 genes)**	*CADPS2, CSPG5, DNAJC6, DOCK1, GABRA1, GABRB2, GRIK1, GRIK4, GRIN2A, GRIN3B, GABRR1, ITSN1, KCNMB4, LPAR1, LRRK2, NPY1R, PLD1, RPH3A, SEPTIN5, SLC6A9, SLC17A6, SLC18A2, STON2, SV2B, TMEM163*
**Regulation of presynaptic membrane potential**	*GABRA1, GABRR1, GABRB2, GRIK1, GRIK4, GRIN2A, GRIN3B, KCNMB4*
**Synaptic vesicle cycle**	*CADPS2, CSPG5, DNAJC6, DOCK1, ITSN1, LPAR1, LRRK2, NPY1R, PLD1, SEPTIN5, RPH3A, SLC17A6, SLC18A2, STON2, SV2B, TMEM163*
**Postsynapse (total 23 genes)**	*ANO6, CACNG4, CACNG5, CNIH2, CRTC1, EFNB2, ERBB2, ERBB4, GABRA1, GABRB2, GLRA2, GPC4, GRIN2A, GRM1, GRIK1, ITPR1, LPAR1, MAGI2, NETO1, NSMF, SNAP23, SYNPO, WNT5A*
**Synaptic signalling (total 32 genes)**	*ADRA2A, ADRA1A, BDNF, CBLN2, CDH11, CHRNA6, DRD1, EFNB3, EPHA7, GABRR1, GLRA3, GPER1, GRM3, GRM8, HTR2A, IGF1, LRRC4, NCAM1, NPY1R, NTNG1, NTRK2, PENK, PLAT, PTN, ROR2, SYNPO, TENM1, TENM2, TENM4, TNR, WNT5A, WLS*
**Transsynaptic signalling**	*ADRA1A, ADRA2A, BDNF, CBLN2, CDH11, CHRNA6, DRD1, EFNB3, EPHA7, GABRR1, GLRA3, GPER1, GRM, GRM8, HTR2A, IGF1, LRRC4, NCAM1, NTNG1, NTRK2, PLAT, PTN, ROR2, SYNPO, TENM1, TENM2, TENM4, TNR, WNT5A, WLS*
**Chemical synaptic transmission**	*ADRA1A, ADRA2A, CBLN2, CDH11, CHRNA6, DRD1, EPHA7, GABRR1, GLRA3, GPER1, GRM3, GRM8, HTR2A, IGF, LRRC4, NCAM1, NTNG1, PTN, ROR2, SYNPO, TNR, WNT5A*
**Synapse organization (total 40 genes)**	*AGRN, CBLN2, CDH6, CDH10, C1QL1, CTTNBP2, CYFIP1, DISC1, DRD1, DLG5, EPHA7, EFNB2, EPHA4, ERBB4, FLNA, FZD1, GPC4, GRN, ITSN1, LAMB2, LRRC4, LRRTM3, MAGI2, MDGA1, NCAM1, NTN1, NTNG1, NTRK3, PTPRO, ROR2, SIPA1L1, SPARC, SPARCL1, SPTB, SYDE1, TANC1, TENM1, TENM2, TENM4, WNT5A*

Supplementary Table 5. Overview on synaptic phenotype-associated significantly underexpressed genes. Synaptic GO terms (synaptic function) in hierarchical structure with associated significantly underexpressed genes in Patient 1 vs CRISPR P1 and all Patients vs Control 1, respectively.
