# Supplementary material for: Neurodevelopmental and synaptic defects in DNAJC6 parkinsonism, amenable to gene therapy
Source: Brain. 2024 Jan 18;147(6):2023–37. doi: 10.1093/brain/awae020 (PMC11146427; doi:10.1093/brain/awae020)
Supplement: awae020_Supplementary_Data [file awae020_supplementary_data.zip › brain-2023-01299-File011.pdf]

Neurodevelopmental and synaptic defects in *DNAJC6* parkinsonism, amenable to gene therapy

Lucia Abela,[1] Lorita Gianfrancesco,[1] Erica Tagliatti,[2,3] Giada Rossignoli,[1] Katy Barwick,[1] Clara Zourray,[1,2] Kimberley M. Reid,[1] Dimitri Budinger,[1] Joanne Ng,[1,4] John Counsell,[1] Arlo Simpson,[1] Toni S. Pearson,[5,6,7] Simon Edvardson,[8] Orly Elpeleg,[8] Frances M. Brodsky,[9] Gabriele Lignani,[1,2] Serena Barral[1] and Manju A. Kurian[1,10]

Author affiliations:

1 Department of Developmental Neurosciences, Zayed Centre for Research into Rare Disease in Children, UCL Great Ormond Street Institute of Child Health, London, WC1N 1DZ, UK

2 Department of Clinical and Experimental Epilepsy, UCL Queen Square Institute of Neurology, University College London, London, WC1N 3BG, UK

3 Laboratory of Pharmacology and Brain Pathology, Humanitas Clinical and Research Center, Via Manzoni 56, 20089 Milano, Italy

4 Gene Transfer Technology Group, UCL-Institute for Women's Health, London, WC1E 6AU, UK

5 Department of Neurology, Columbia University Irving Medical Center, New York, NY 10032-3784, USA

6 Department of Pediatrics, Nationwide Children's Hospital, Ohio State University, Columbus, OH 43210, USA

7 Department of Neurology, Nationwide Children's Hospital, Ohio State University, Columbus, OH 43210, USA

8 Department of Genetics, Hadassah, Hebrew University Medical Center, 9574869 Jerusalem, Israel

9 Research Department of Structural and Molecular Biology, Division of Biosciences, University College London, London WC1E 6BT, UK

10 Department of Neurology, Great Ormond Street Hospital, London WC1N 3JH, UK

Supplementary Materials and Methods:

Generation of isogenic control by CRISPR/Cas9 gene editing

Generation of a CRISPR/Cas9-corrected iPSCs line for Patient 1 was carried out by Applied StemCell Inc. (Milpitas, CA). In brief, two single guide RNAs (sgRNA) were designed to target the *DNAJC6* genomic locus and tested in HEK293 cells for Cas9-mediated cleavage efficiency. Patient 1 iPSCs were subsequently co-transfected (Neon transfection system, Invitrogen) with sgRNAs and a single-stranded oligo donor (ssODN) for endogenous homology repair. Single cells were culture in 96-well plates for 14 days and subsequently transferred to 24-well plates for further expansion. Genomic DNA was extracted from each clone to confirm via PCR bi-allelic correction of the homozygous *DNAJC6* mutation.

DNAJC6 direct Sanger sequencing in patient-derived iPSCs

gDNA was extracted using the DNeasy Blood & Tissue kit (QIAGEN) following manufacturer instructions. Primers were designed with Genomic DNA sequences were from Alamut® Visual 2.11 software (Genome Reference NM_001256864.2). Primers were designed with Primer3Plus software (http://www.bioinformatics.nl/cgi-bin/primer3plus/primer3plus.cgi). PCR conditions and Primer sequences are available on request. PCR products were checked on a 1.5% agarose gel and subsequently purified with MicroCLEAN Kit (Clent Life Science) and processed for sequencing with the BigDye® Terminator v1.1 Cycle Sequencing Kit (Thermo Fisher Scientific). Sequencing reactions were run on the ABI PRISM 3730 DNA 27 Analyzer (Applied Biosystems) and results were analysed with Sequencher (https://www.genecodes.com) and Chromas software (http://technelysium.com.au/wp/chromas).

Karyotyping with single-nucleotide polymorphism (SNP) Array

Genome integrity was assessed by SNP array analysis performed by UCL Genomics on genomic DNA extracted from iPSCs. Infinium HumanCytoSNP-12 v2.1 BeadChip array was the software used and the data were analysed with Bluefuse Multi software (Illumina).

Analysis of pluripotency by Epi-Pluri Score

Genomic DNA was extracted from iPSCs pellets using the DNeasy Blood & Tissue kit (QIAGEN). The Epi-Pluri-Score analysis was performed by Cygenia, Epigenetic Diagnostics,

Aachen, Germany. The epigenetic pluripotency marker is based on the combination of DNA methylation levels at three specific CpG sites located within two genes: *ANKRD46* (methylated in pluripotent cells) and *C14orf115* (non-methylated in pluripotent cells)[1].

Analysis of pluripotency by *in vitro* spontaneous differentiation assay

For Patient 1 iPSCs lines, two wells were harvested with TriplE and resuspended in KOSR medium with Thiazovivin (Cambridge Biosciences) in a nonadherent bacterial dish (6 cm^2) to form embryoid bodies (EB). Media was changed on day 2. On day 4, EBs were plated on plates previously coated with gelatine 0.1% (Sigma-Aldrich) for mesoderm differentiation or Matrigel for ectoderm and endoderm differentiation. Differentiations were cultured for 16 days with media change every other day. The gelatine-coated wells were fed with DMEM medium supplemented with 20% FCS, while the Matrigel-coated wells were fed with KOSR medium. For Patient 2 and 3 spontaneous *in vitro* differentiation was performed according to manufacturer instructions from the STEMdiff™ Trilineage Differentiation Kit (StemCell Technologies).

Immunocytochemistry

Cells were washed with DPBS (Invitrogen) and fixed for 10 minutes at room temperature (RT) with 4% paraformaldehyde (PFA). The cells were then incubated in blocking solution (PBS, 10% FBS, 0.1% Triton-X100 [Sigma]) for 30 min, followed by incubation of antibodies overnight at 4 °C. Primary antibodies for pluripotency markers were used in following dilutions: OCT4 (1:50, Santa Cruz), NANOG (1:500, Millipore), TRA-1-60 (1:200, Santa Cruz), TRA-1-81 (1:200, Millipore). Blocking solution did not contain Triton for the cell surface markers TRA-1-60 and TRA-1-81. Blocking buffer for immunostaining at day 65 contained Triton 0.3%. Please see Supplementary Table 1 for all primary antibodies. The next day, cells were washed 3x with PBS and incubated with secondary antibodies in blocking solution in the dark at RT for 45min. Secondary antibodies were used in following dilutions: Alexa Fluor® 594 Goat Anti-mouse IgG (1:400; Alexa Fluor, Life Technologies), Alexa Fluor® 488 Goat Anti-mouse IgG (1:400, Alexa Fluor, Life Technologies). DAPI was used in a concentration of 1:1,000 for nuclear staining. Imaging was performed on the Olympus IX71 inverted TC scope for assessment of pluripotency and spontaneous in vitro differentiation in iPSCs, and Day 11 mDA precursors. All other immunocytochemistry studies were performed on a multiphoton confocal microscope (Zeiss LSM880) and the images were processed using ImageJ software

(National Institutes of Health). Total quantification was performed on 3 or more random fields from each independent experiment and a total of 1800 randomly selected nuclei (FOXA2/LMX1A, TH/MAP2, cCAS3), and 1200 nuclei respectively (NeuN), were counted to extract the final data. Analysis of primary neurite branching was analyzed as an average from 150 dispersed nuclei in random fields from 4 different biological replicates.

Reverse transcription PCR (RT-PCR) and Quantitative Real Time PCR (qRT-PCR)

RNA was purified using the RNeasy mini kit (Qiagen) according to manufacturer instructions. After elimination of double strand DNA contaminants using DNAseI purification kit (Invitrogen), cDNA was produced by reverse transcription using Superscript III (Invitrogen). Sendai virus clearance was assessed via PCR carried out according to instructions from the CytoTune^{TM}-iPS Sendai Reprogramming Kit. qRT-PCR analysis was performed using StepOnePlus^{TM} Real-Time PCR System (Applied Biosystems). Reaction mix was prepared with 1x MESA Blue qPCR MasterMix Plus for SYBR® Assay (Eurogentec), 1:25 cDNA and 500 nM of each primer. The protocol used is denaturation of 95°C for 5 min, followed by 40 cycles of 15 seconds denaturation at 95°C and 1 min annealing/extension at 60°C. Relative quantification of gene expression was determined using the $2^{-\Delta\Delta Ct}$ method with *GAPDH* as housekeeping reference gene, and normalized to the corresponding iPSCs.

Total protein extraction and quantification

Total proteins were extracted from the cells using ice-cold RIPA lysis and extraction buffer (Sigma-Aldrich) supplemented with protease inhibitors cocktails (Roche). After 30 min incubation on ice, the mix was centrifugated at 13.000×*g* for 15 min and the supernatant was then collected. Total proteins were quantified with Pierce™ BCA Protein Assay Kit (Thermo Fisher Scientific) using provided standards.

Immunoblotting

10, or 30 µg of protein, respectively, was denatured with Laemmli buffer (Bio-Rad Laboratories LTD) with dithiothreitol (DTT). Proteins were separated with Mini-PROTEAN TGX Stain Free Gels (Bio-Rad Laboratories LTD) and transferred to a Trans-Blot Turbo Transfer membrane (Bio-Rad Laboratories LTD). The membranes were blocked in in 5% milk, 1x TBS, 0.1% Tween for 1h at room temperature and subsequently incubated with primary antibodies (Supplementary Table 1) at 4°C overnight. Membranes were then incubated with the secondary

anti-rabbit or anti-mouse horseradish peroxidase-conjugated antibody at a dilution of 1:3000 (Cell Signalling). Immunoreactive proteins were visualized with Chemidoc MP (Bio-Rad Laboratories). Beta-actin was used as a control for equal protein loading and to quantify the total amount of endogenous protein. The membranes were reprobed after clearance with Restore Western Blot Stripping Buffer (Thermo Scientific) to identify proteins with similar weight. The intensity of immunoreactive bands was analyzed using ImageJ software (National Institutes of Health). The density of the bands was normalized to the loading controls.

Electrophysiological recordings of mDA neurons

For electrophysiological recordings of sEPSCs, day 70 mDA neurons plated on glass coverslips coated with laminin and poly-ornithin were transferred to a recording chamber and continually perfused with room temperature, carbogenated ACSF containing (in mM): 125 NaCl, 2.5 KCl, 25 NaHCO3, 1.25 NaH2PO4, 25 glucose, 1 MgCl2, 2 CaCl2). Whole-cell patch-clamp recordings were performed using thin-walled borosilicate glass pipettes (1.5 mm OD, 1.17 mm ID; Harvard Apparatus).

Pipettes had a tip resistance of 4-5 MΩ when filled with a K-gluconate based internal solution (in mM: 142 K-gluconate, 4 KCl, 0.5 EGTA, 10 HEPES, 2 MgCl2, 2 Na2ATP, 0.3 Na2GTP, 1 Na2Phosphocreatine, pH = 7.25, 285-295 mOsm). All the recordings were carried out from neurons held at -70mV at room temperature with continuous perfusion of the extracellular solution. Electrophysiological recordings were made with a Multiclamp 700A amplifier (Axon Instruments, Molecular Devices). The amplifier was used in combination with Signal 6.0 software (Cambridge Electronic Design, Ltd). The data were filtered at 10 kHz and digitized at 50 kHz (BNC-2090, NI-6221, National Instruments). sEPSCs were recorded for 250-300s, of which 200s were used for analysis. Standardised, semi-automatic detection of events was performed using ClampFit (Molecular Devices). A calculated +10mV liquid-junction potential was corrected for and added to the RMP values.

Lentivirus production and gene delivery

The lentiviral expression plasmid (pCCL-hSYN-DNAJC6-IRES-EGFP) was developed by inserting the human *DNAJC6* gene coding (NM_001256864.2) into a plasmid kindly provided by Dr Joanne Ng (UCL, Institute of Women's Health, UCL), who also developed the control plasmid CCL-hSYN-GFP. Sanger sequencing was used to verify the plasmid sequences. Primer sequences and PCR conditions are available upon request. HEK293T were used to produce the lentiviral vectors and the same cell type was transduced to validate the lentiviral expression

plasmid performing immunoblotting for auxilin 7 days later post-transduction. Lentiviral vectors were produced using a 2nd generation packaging system[2] and vector titers were quantified by qPCR as previously described[3] using primers listed in Supplemental Table 2. mDA neurons were differentiated for 28 days before transduction with lentiviral vectors at 1.5 multiplicity of infection for 24 h, and subsequently differentiated, as previously described to day 70.

References

1. Lenz M, Goetzke R, Schenk A, et al. Epigenetic biomarker to support classification into pluripotent and non-pluripotent cells. Sci Rep. Nature Publishing Group; 2015;5:8973.
2. Counsell JR, Asgarian Z, Meng J, et al. Lentiviral vectors can be used for full-length dystrophin gene therapy. Sci Rep. Sci Rep; 2017;7.
3. Barczak W, Suchorska W, Rubiś B, Kulcenty K. Universal real-time PCR-based assay for lentiviral titration. Mol Biotechnol. Mol Biotechnol; 2015;57:195–200.
